# AFM reveals differential effects of acidification on LDL– and oxidized LDL–receptor interactions: biomechanical implications in atherogenesis

**DOI:** 10.1186/s11658-025-00715-9

**Published:** 2025-03-18

**Authors:** Kun Wang, Chenhan Sun, Hongda Zhuang, Xian-Cheng Jiang, Yong Chen

**Affiliations:** 1 School of Life Sciences, Nanchang University, 999 Xuefu Ave., Honggutan District, Nanchang, 330031 Jiangxi People’s Republic of China; 2 Institute for Advanced Study, Nanchang University, Nanchang, 330031 Jiangxi China; 3 Department of Cell Biology, SUNY Health Science University, State University of New York, Brooklyn, NY 11203 USA; 4 School of Pharmacy, Nanchang University, Nanchang, 330031 Jiangxi China

**Keywords:** Atomic force microscopy (AFM), Low-density lipoprotein (LDL), Atherosclerosis

## Abstract

The receptor recognition and interaction of plasma lipoproteins (e.g., native low-density lipoproteins (LDL)/oxidized low-density lipoproteins (oxLDL), as well as the influence of microenvironmental/lysosomal acidification, play critical roles in lipoprotein metabolism and diseases (e.g., atherosclerosis) but have been less investigated. Here, the recognition/interaction of LDL or oxLDL with LDL receptor (LDLR) or CD36 (a scavenger receptor) or with living cells at various pHs was evaluated mainly via atomic force microscopy (AFM). To improve force measurement accuracy, a novel, micro-droplet-based method for AFM probe functionalization was developed. We found that solution acidification significantly reduced the LDL–LDLR binding at pH ≤ 6.4, whereas the oxLDL–CD36 binding had no significant change until pH ≤ 4.4. Compared with a traditional immersion method, our micro-droplet method for AFM probe functionalization produced more accurate interaction forces, and revealed that acidification significantly reduced the LDL–LDLR/cell interaction forces, instead of the oxLDL–CD36/cell-specific interaction forces and nonspecific interaction forces. The data imply that the LDL–LDLR/cell recognition and interaction are susceptible to acidification, whereas the oxLDL–CD36/cell recognition and interaction are tolerant of acidification. The results may provide important novel information and biomechanical/pathological implications for understanding lipoprotein metabolism and atherosclerosis.

**Supplementary Information:**

The online version contains supplementary material available at 10.1186/s11658-025-00715-9.

## Introduction

Plasma lipoproteins are the major transporters of lipids in the blood circulation of animals, particularly of human beings, mainly including chylomicron (CM), very-low-density lipoprotein (VLDL), intermediate-density lipoprotein (IDL), low-density lipoprotein (LDL), and high-density lipoprotein (HDL) [1]. When these lipid transporters reach their destinations (organs/tissues/cells), they will be recognized and bound by the extracellular parts of specific receptors in the plasma membrane of cells, which then triggers the downstream steps inside cells. Therefore, measuring the recognition and interaction force between plasma lipoproteins and their corresponding receptors is vital for understanding the functions and underlying mechanisms of different lipoproteins. Until now, unfortunately, the interaction forces between plasma lipoproteins and their corresponding receptors are poorly understood.

Plasma lipoproteins not only deliver lipids to all cells, providing beneficial lipid molecules for cellular construction and metabolism, but also are related with many diseases, including cardiovascular diseases (CVD), particularly atherosclerotic CVD (ASCVD). It is well known that both LDL cholesterol (LDL-C) and HDL cholesterol (HDL-C) are closely correlated with atherosclerosis. The majority of studies support the positive and negative roles of LDL and HDL, respectively, in the initiation and progression of atherosclerosis [2, 3]. Many forms of LDL, including modified LDL (particularly oxidized LDL [oxLDL]) [4, 5], small dense LDL (sdLDL) [5, 6], aggregated native LDL (agLDL) [7, 8], and others are reported to initiate or aggravate atherosclerosis. The receptor for native LDL (i.e., LDL receptor [LDLR]) is different from those of modified LDL types (e.g., oxLDL); they have their own specific receptors (i.e., scavenger receptors [SRs]), whose family includes more than 10 classes (A–J) [9, 10], such as CD36 in class B for oxLDL recognition (CD36 mainly recognizes oxidized phospholipids on oxLDL particles).

After recognition by corresponding receptors at the cell surface, LDL or oxLDL will be internalized into endolysosomes or lysosomes in which LDL or oxLDL will enter an acidic environment of down to pH ~4.5 [11]. Moreover, an extracellular acidic microenvironment of down to pH ~5.5 has long been reported in many pathological tissues, such as advanced atherosclerotic lesion, solid tumors, synovial fluid/membrane in joint diseases, and others [11]. Atherosclerotic lesions are often hypoxic with elevated lactate concentrations and local acidification of extracellular fluids [12, 13]. In these acidic microenvironments, the recognition and interaction between LDL/oxLDL and their receptors also occur frequently. Then, a scientific question about whether or how an acidic microenvironment influences the recognition and interaction between LDL/oxLDL and their specific receptors may arise. In our previous study, we have provided evidence supporting that acidic conditions can significantly affect the physical properties (e.g., size, stiffness, and stickiness) of LDL/oxLDL particles [11]. Therefore, we hypothesized that an acidic microenvironment can potentially influence the biological fates of LDL/oxLDL and the pathological progression of atherosclerosis by differentially altering the receptor recognition and interaction of LDL/oxLDL. In the present study, this hypothesis was tested mainly by applying the imaging and force spectroscopic functions of atomic force microscopy (AFM) on a nanometer or piconewton scale.

## Materials and methods

### Reagents and cell culture

Human low-density lipoprotein (LDL) and oxidized LDL (oxLDL) were purchased from Yiyuan Biotechnologies (Guangzhou, China; Cat. No. YB-001 and Cat. No. YB-002 for LDL and oxLDL, respectively). Native LDL was isolated from blood-bank-produced human plasma and purified to homogeneity via ultracentrifugation (1.019–1.063 g/cc). oxLDL was produced by copper-induced oxidation in phosphate-buffered saline (PBS) at 37 °C for 18 h, and verified by us using agarose gel electrophoresis (a more than twofold faster migration than native LDL) and the thiobarbituric acid reactive substances (TBARS) method (malondialdehyde [MDA] ~0.24 nmol/mg protein for native LDL and MDA~22.2 nmol/mg protein for oxLDL). This type of oxLDL can induce the formation of foam cells and therefore is widely used to study lipid metabolism and atherosclerosis.

Recombinant human CD36, LDL receptor (LDLR), and anti-CD36 and anti-LDLR antibodies were from Abcam (Cambridge, MA). *N*,*N*-Diisopropylethylamine (DIPEA) and aminopropyltrethoxysilane (APTES) for basic modification of micas were from Xiya Reagent (Chengdu, China). Glutaraldehyde (25%) for basic modification of micas was from Sigma. Carboxyl colloidal Au nanoparticle (5 nm in diameter) was from Nanoeast Biotech Co., Ltd. (Nanjing, China). AFM probes were from Nanosensors (Swiss Confederation).

Human umbilical vein endothelial cells (HUVECs; Cat No. FH1122) were purchased from Shanghai Fuheng Biotechnology Co., LTD (Shanghai, China). The cells were cultivated routinely in Roswell Park Memorial Institute (RPMI) 1640 medium (Sigma) supplemented with 10% (*w*/*v*) fetal bovine serum and 1% penicillin–streptomycin solution (Solarbio Science & Technology Co., Shanghai, China), which contains 100 U/mL penicillin and 100 μg/mL streptomycin. The cells at passage ~5 and at ~75% confluency were used for AFM experiments. Prior to AFM experiments, the cells were cultured in a medium (pH 7.4) supplemented without fetal bovine serum for 6 h. During AFM imaging, the medium was replaced by an acidic solution at indicated pH, and the cells were under the indicated acidic condition for less than 0.5 h. The MTT assay for the viability of HUVECs under different acidic conditions for 6 h (Fig. S1 in Supplementary Information) indicates that HUVEC cells could survive for at least 6 h at pH > 5.5, and over 70% of cells still can survive at pH 4.5. In our experiments, the cells stayed in an acidic solution for a much shorter period of time (generally less than 0.5 h). Therefore, most HUVEC cells can survive through this short period of time at pH 4.4 in the present study.

### Basic modification and sample functionalization of mica

The method for basic modification of micas (APTES–micas) was modified from our previous studies [11, 14]. Briefly, a clean and dry glass dessicator was purged with ultrapure argon to remove the air and moisture; then, freshly cleaved mica sheets (sheets of the silicate mineral mica) in clean petri dishes and two containers without lids containing 400 μL of DIPEA and 200 μL of APTES, separately, were immediately put inside the dessicator successively, which was purged with argon for ~2 min in between; after the mica sheets were exposed to APTES vapor for ~4 h, the APTES container was removed, and the dessicator was purged with argon and sealed for more than 4 days. Prior to AFM experiments, the mica sheets were taken from the dessicator, immediately incubated with 1 mL of 0.2% fresh glutaraldehyde solution for ~1 h, and washed with double distilled water. For sample preparation for AFM imaging, the mica sheets after basic modification were immediately incubated with 100 μg/mL receptor samples (i.e., LDLR or CD36) in PBS at pH 7.4 for ~1 h, washed with PBS, and incubated with 0.2 mg/mL of corresponding lipoproteins (i.e., LDL or oxLDL, respectively) at an indicated pH (i.e., pH 7.4, pH 6.4, pH 5.4, and pH 4.4) for ~1 h. After removing excess lipoprotein solution and washing with PBS at the corresponding pH, the mica sheets were then subjected to AFM measurement in PBS buffer at the corresponding pH. For sample preparation for AFM force spectroscopy, the mica sheets after basic modification were immediately incubated with 0.2 mg/mL lipoprotein samples (i.e., LDL or oxLDL) for ~1 h, washed with PBS at pH 7.4, and then subjected to AFM measurement in PBS buffer at different pH values (i.e., pH 7.4, pH 6.4, pH 5.4, and pH 4.4) by using functionalized AFM probes as described below.

### AFM probe functionalization of samples via our micro-droplet method

The method for basic modification of AFM probes was modified from a previous report [15]. Briefly, AFM probes were washed first in chloroform and then in piranha solution (98% H_2_SO_4_:30% H_2_O_2_ :=  7:3; *v*/*v*) for 30 min. After air drying in a fume hood for 30 min, the probes were washed with water, dried with nitrogen flow, washed twice with chloroform, and dried again with nitrogen flow. Then, the probes were exposed in air for more than 30 min to form a thin layer of silicon oxide with a silanol group (Si–OH) on the probe surface, washed three times with chloroform, and dried with nitrogen flow. Next, the probes were incubated with 1% mercaptopropyltrimethoxysilane (MPTMS)/toluene (*v*/*v*) solution for 2 h and successively cleaned by toluene, acetone, and ethanol.

For AFM probe functionalization of samples via our micro-droplet method, a solution containing 3 mg/mL Maleimide-polyethylene glycol-*N*-hydroxysuccinimide (MAL-PEG-NHS) was prepared and sprayed on the surface of a hydrophobic glass coverslip to make a layer of micro-droplets on the glass coverslip. After putting an AFM probe with basic modification and the glass coverslip with micro-droplets in an AFM system, the tip of the AFM probe was located above a micro-droplet with a relatively small size (< 50 μm in diameter) and moved toward the micro-droplet; after contacting the micro-droplet, the probe tip further dipped into the micro-droplet to a certain depth (e.g., ~2 μm in this study) until a deflection of the probe occurred according to the “DEFLECTION” window on the operating interface, and held on for 5 min; then, the probe tip was retracted away from the micro-droplet. The whole process including the approaching, dipping, and retraction, which was controlled by a stepping motor equipped in the AFM system, was repeated 2–3 times. Subsequently, the probe was taken away from the AFM system, washed with chloroform, and dried with nitrogen flow (then, a probe tip with MES-PEG-NHS modification was prepared). Similarly, a solution mixing 10 mM LDLR or CD36 in double distilled water with 2 μg ethylene diamine tetraacetic acid (EDTA), 5 μL hydroxyethyl piperazine ethanesulfonic acid (HEPES), and 5 μL trichloroethyl phosphate (TCEP) was prepared and sprayed on the surface of a hydrophobic glass coverslip to make a layer of micro-droplets on the glass coverslip. Via the same process as our micro-droplet method, the MES-PEG-NHS-modified probe tip was further modified with LDLR or CD36. After washing three times with PBS buffer (pH 7.4), an AFM probe functionalized with PEG-LDLR/CD36 on the probe tip was prepared via our micro-droplet method and immediately subjected to AFM force spectroscopy or stored at 4 ℃ for less than 2 days.

For AFM probe functionalization of samples via a traditional immersion method, AFM probe was immersed in the solution containing 3 mg/mL MES-PEG-NHS for 60 min, washed with chloroform, and dried with nitrogen flow. Next, this probe with the MES-PEG-NHS modification was immersed in the solution mixing 10 mM LDLR or CD36 in double-distilled water with 2 μg EDTA, 5 μL HEPES, and 5 μL TCEP for 60 min. After washing three times with PBS buffer (pH 7.4), the AFM probe functionalized with PEG-LDLR/CD36 was prepared via the traditional immersion method and immediately subjected to AFM force spectroscopy.

### AFM topographical imaging and force spectroscopy in buffer at various pHs

An Agilent Series 5500 AFM (Agilent Technologies, CA) equipped with a scanner of 9 μm × 9 μm × 2 μm for topographical imaging and lipoprotein–receptor interaction or with a scanner of 90 μm × 90 μm for lipoprotein–cell interaction was utilized. The samples were detected by silicon nitride tips (qp-BioAC; NanoSensors, USA) with an end radius of ~10 nm and a cantilever spring constant of ~0.06 N/m, which was measured by the thermal K method (i.e., thermal tune method) program equipped with the AFM instrument. Tapping mode and contact mode were used for topographical imaging in liquid (scan speed: 0.5 Hz) and for lipoprotein–receptor/cell interactions (loading force: 0.5 nN for 2 s; loading speed: 200 nm/s), respectively, at 37 °C. A Cypher VRS1250 AFM (Oxford Instruments, CA) equipped with a scanner of 30 μm × 30 μm × 5 μm was also used for topographical imaging (e.g., the observation of the recognition of a native LDL by the LDL receptor at pH 7.4, one of which was pretreated at different pHs). All topographical images (scan range: 1 μm × 1 μm; image resolution: 512 × 512) were flattened at most by one level. Interaction force was extracted from the retraction curves of force-versus-distance curves via PicoView 1.14 software equipped with the AFM instrument. The curves with only one peak were selected to extract the interaction forces for the statistical quantification of the force. PBS buffers at various pHs were prepared by using hydrochloric acid to adjust the pH value of a buffer. A simple homemade microfluidic system was utilized to dynamically change the solutions at various pHs on the AFM sample stage in a specific experiment.

### Gold nanoparticle–receptor conjugation and immuno-transmission electronic microscopy (Immuno-TEM)

Approximately 1 mL of carboxylated gold nanoparticles (5 nm in diameter; Nanoeast Biotech, Nanjing, China) were mixed and incubated with 50 μL of 15 mM 2-(*N*-morpholino) ethanesulfonic acid (MES) solution and 50 μg LDLR/CD36 at room temperature for 1 h. After an ultrasonic oscillation, the solution was mixed with 50 μL of 10 mg/mL 1-ethyl-3-(3-dimethylaminopropyl) carbodiimide (EDC) coupling buffer freshly prepared, ultrasonicated at room temperature for 15 min, and incubated for 2 h on a shaking table. Then, 50 μL of 5 mg/mL bovine serum albumin (BSA) solution was added to terminate the coupled reaction at 37 °C for 1 h. After a centrifugation at 20,000 × *g* for 15 min and washing three times with PBS, the gold-nanoparticle-conjugated receptor molecules were stored at 4 °C.

For transmission electronic microscopy (TEM) observation, 50 μL of 0.2 mg/mL LDL was incubated on a shaking table with 10 μL of the pre-prepared gold-nanoparticle-conjugated receptor molecules (LDLR or CD36) at 37 °C for ~8 h. After a centrifugation at 10,000 × *g* for 30 min, the supernatant was centrifuged twice in an ultrafiltration centrifuge tube of 100 kDa at 20,000 × *g* for 30 min. After dissolving in 100 μL PBS, 1 μL of the samples were transferred onto 300 mesh copper grids for 10–60 s, gently rinsed three times with pure water, gently rinsed three times with 10 μL of 1% uranyl acetate, and incubated with 1 μL of 1% uranyl acetate for 5 min. After removing the staining solution but with a thin layer of solution remaining, the copper grids with samples were dried in a desiccator for more than 8 h, and subjected to TEM imaging.

### Statistical analysis

The data in the text and graphs are expressed as mean ± SD. For all analyses, normal distribution was tested using the Shapiro–Wilk normality test. Statistical analyses are performed using one-way analysis of variance (ANOVA) among multiple groups (Tukey’s multiple comparisons test) or Student *t* test between two groups (Welch’s correction for unequal variances was performed) to determine the significance (*p* < 0.05 is regarded as a significant difference), which is presented in the figure legend.

## Results

### The specific receptor recognition of native/oxidized LDL particles at various pHs imaged by AFM and immuno-TEM

**Fig. 1:**
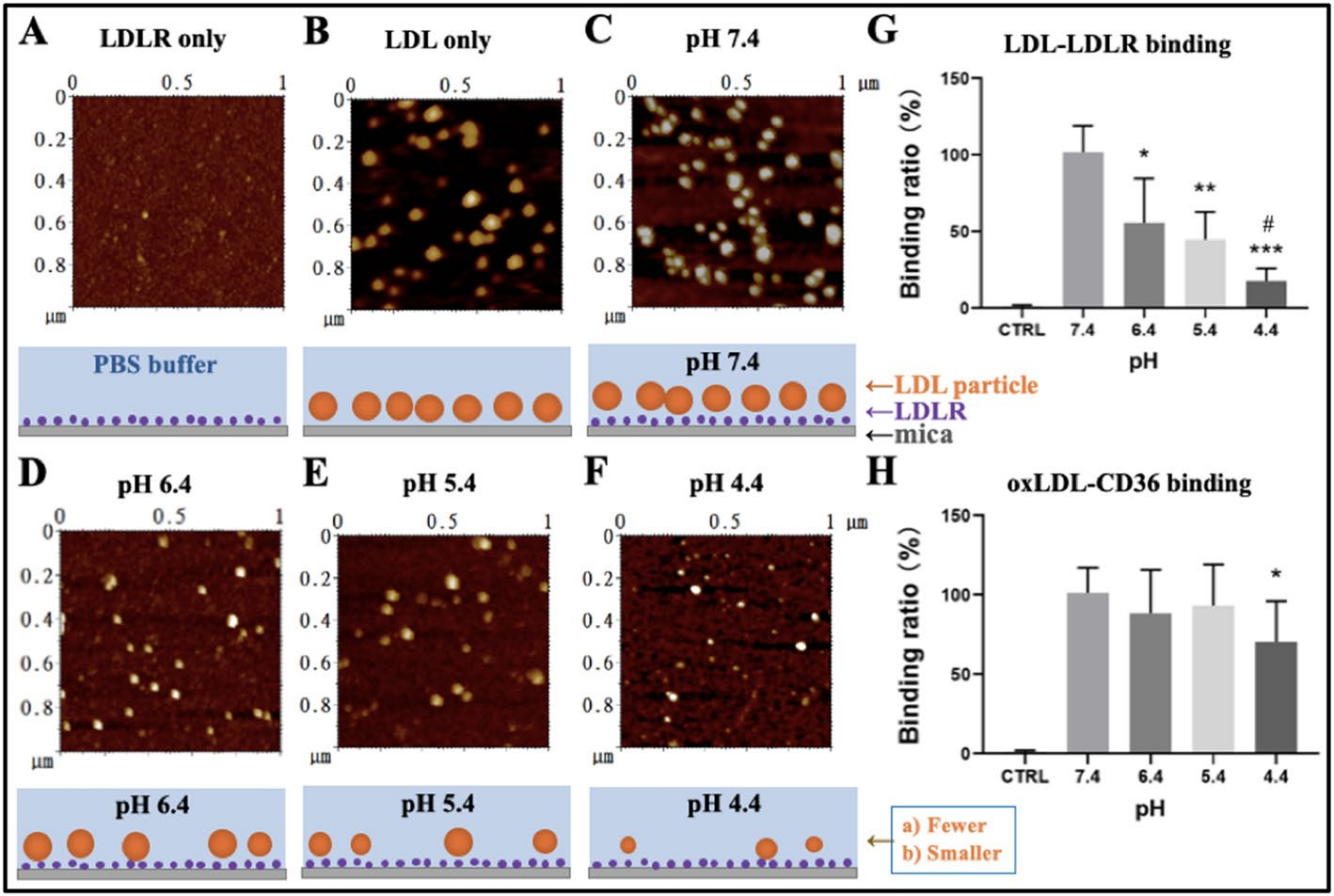
AFM topographical observation of the recognition of native or oxidized LDL by receptors (LDLR or CD36) pre-immobilized on micas. **A** LDLR only. **B** Native LDL particles only. **C**–**F** Binding of native LDL particles to LDLRs at pH 7.4, pH 6.4, pH 5.4, and pH 4.4, respectively. **A**–**F** Top panels: AFM topographical images; bottom panels: Schematic diagrams presenting the binding of LDL particles (brown) onto LDLR molecules (purple) pre-immobilized on mica (gray). AFM imaging was performed in PBS buffer at different pH values. **G** Quantitative analysis of the LDL–LDLR binding ratio at different pH values. **H** Quantitative analysis of the oxLDL–CD36 binding ratio at different pH values (the representative AFM topographical images are not shown). The control group (Ctrl) means the group incubating of LDL/oxLDL on a mica surface coated without receptors to exclude the possibility of nonspecific interaction. ^*^p < 0.05, ^**^p < 0.01, and ^***^p < 0.001 compared with pH 7.4; ^#^*p* < 0.05 compared with pH 6.4 (*n* ≥ 3 images in each group)

Previously, by using the imaging function of atomic force microscopy (AFM), we have visualized single particles of various plasma lipoproteins including high-density lipoprotein (HDL), low-density lipoprotein (LDL), oxidized LDL (oxLDL), acetylated LDL (acLDL, another type of modified LDL), and very-low-density lipoprotein (VLDL); measured their sizes; and detected their recognition/binding by CD36 and SR-B1, two class B scavenger receptors at pH 7.4 [16]. By using the force spectroscopic function of AFM, we also detected the changes in size, stiffness, and stickiness of LDL and oxLDL particles upon solution acidification in another previous study [11, 17, 18]. However, the acidification-induced changes of the receptor recognition/interaction of LDL/oxLDL have never been investigated via AFM. Here, the LDL–LDLR and oxLDL–CD36 bindings/recognitions were detected by the imaging function of AFM (Fig. 1). The receptors (LDLR or CD36) were pre-immobilized on basically modified micas, then incubated with lipoprotein particles (LDL or oxLDL) in solutions at various pH values (pH 7.4, 6.4, 5.4, and 5.4, respectively), and imaged by AFM in PBS after PBS washing. The representative AFM topographical images of oxLDL particles binding on the CD36 receptor layer are presented in Fig. S2 in Supplementary Information.

According to the AFM topographical mapping, the LDLR receptor molecules, which are tiny dots in the AFM image (Fig. 1A), are much smaller than LDL particles (Fig. 1B), which has a size of around 15–30 nm (we did not calculate the average diameter of LDL/oxLDL particles in this study). When LDL particles are bound onto the LDLR layer, LDL particles can be clearly distinguished from the LDLR layer (Fig. 1C), which helps the quantification of LDLR-binding LDL particles for the calculation of the LDL binding ratio. Even based on the mapping, it is obvious that, compared with the pH 7.4 group (Fig. 1C), both the size and amount of LDL particles decrease under acidic conditions (Fig. 1D for pH 6.4, Fig. 1E for pH 5.4, and Fig. 1F for pH 4.4). The changes in size of LDL particles after solution acidification are consistent with our previous report [11]. The statistical analysis (Fig. 1G) shows that solution acidification could significantly reduce the binding ratio of LDL particles in a pH-dependent manner, further confirming the acidification-induced changes in amount of LDL particles in the representative AFM images. AFM also imaged the acidification-induced size decrease of oxLDL (Fig. S2 in Supplementary Information). Similar AFM experiments were performed on the oxLDL–CD36 binding (the representative AFM topographical mapping is not shown here), and the statistical analysis (Fig. 1H) shows that only the acidification of solution at pH 4.4 caused a significant decrease in binding ratio.

**Fig. 2:**
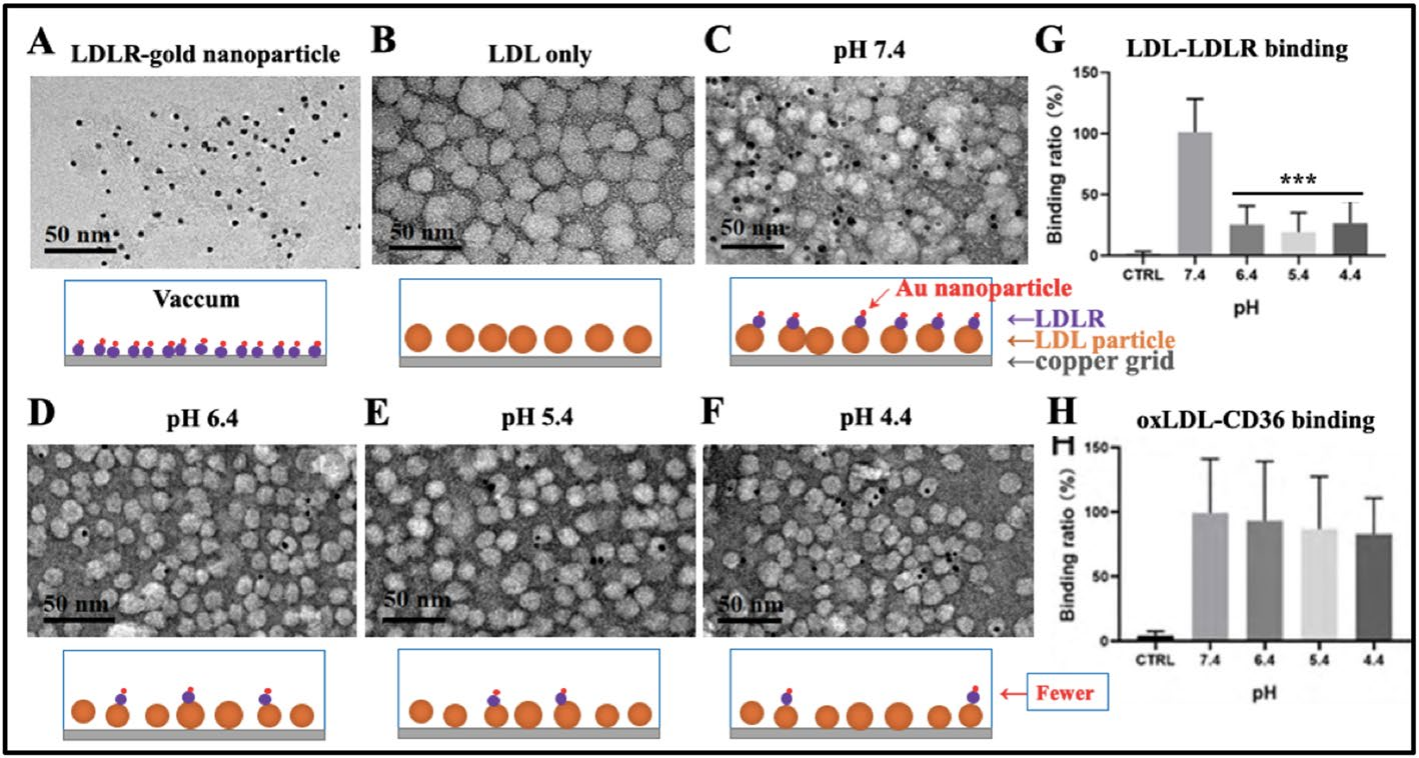
Immuno-TEM observation of the recognition of native or oxidized LDL pre-immobilized on micas by gold-nanoparticle-conjugated receptors (LDLR or CD36). **A** Gold nanoparticle (5 nm)-conjugated LDLR only. **B** Native LDL particles only. **C**–**F** Binding of gold-nanoparticle-conjugated LDLRs to native LDL particles at pH 7.4, pH 6.4, pH 5.4, and pH 4.4, respectively. **A**–**F** Top panels: TEM images; bottom panels: schematic diagrams presenting the binding of gold nanoparticle (red)-conjugated LDLR molecules (purple) onto a LDL particle (brown) layer pre-immobilized on copper grid (gray). LDL–LDLR interaction was conducted in PBS buffer at different pH values, whereas TEM imaging was performed in a vacuum. **G** Quantitative analysis of the LDL–LDLR binding ratio at different pH values. **H** Quantitative analysis of the oxLDL–CD36 binding ratio at different pH values (the representative TEM images of oxLDL–CD36 recognition are not shown). The control group (Ctrl) means the group incubating of gold nanoparticles (but no LDLR) with LDL/oxLDL on micas to exclude the possibility of nonspecific interaction. The number of gold nanoparticles (black dots) in each image was counted for calculation of the binding ratio (to the data at pH 7.4). ^***^p < 0.001 compared with pH 7.4 (*n* ≥ 3 images in each group)

To ensure the specificity of binding measurements, immuno-transmission electron microscopy (immuno-TEM) was also performed (Fig. 2). Gold nanoparticles of 5 nm (black dots in Fig. 2A) were used to label LDLR molecules. LDL particles of ~15–30 nm in diameter were observed by TEM (Fig. 2B), similar to LDL particle sizes imaged by AFM (Fig. 1B). After specific binding of gold-nanoparticle-conjugated LDLR with LDL particles, black dots (i.e., gold nanoparticles) are clearly observed on LDL particles in TEM images (Fig. 2C–F), showing that there are much more black dots in the pH 7.4 group (Fig. 2C) than in the other acidic pH groups (Fig. 2D–F). The statistical analysis of the LDL–LDLR binding ratio (Fig. 2G) also confirms this observation, implying solution acidification could decrease the specific recognition/binding between LDL and its receptor (i.e., LDLR). Moreover, compared with the pH 7.4 group (Fig. 2C), the smaller size of LDL particles was observed in the acidic groups (Fig. 2D–F), which is consistent with the AFM data and with our previous report [11]. Immuno-TEM also imaged the acidification-induced size decrease of oxLDL (Fig. S3 in Supplementary Information). Similar immuno-TEM experiments were performed on the oxLDL–CD36 recognition/binding (the representative TEM images are not shown here), and the statistical analysis of the oxLDL–CD36 binding ratio (Fig. 2H) shows that the acidification of the solution also induced a slight, but not statistically significant, decrease in binding ratio. Therefore, most of the immuno-TEM results coincide with the data from the AFM experiments. The representative immune-TEM images of gold-nanoparticle-conjugated CD36 binding to oxLDL particles are presented in Fig. S3 in Supplementary Information.

Taken together, both AFM and immuno-TEM imaging and statistical analyses show that solution acidification could reduce the particle sizes of LDL/oxLDL and the recognition/binding between LDL/oxLDL and their corresponding receptors (LDLR/CD36) and that native LDL–LDLR recognition/binding is relatively susceptible to solution acidification, whereas oxLDL–CD36 recognition/binding is relatively tolerant of solution acidification.

### Development of a novel, versatile micro-droplet method for AFM probe tip functionalization to improve the accuracy of AFM force spectroscopy

**Fig. 3:**
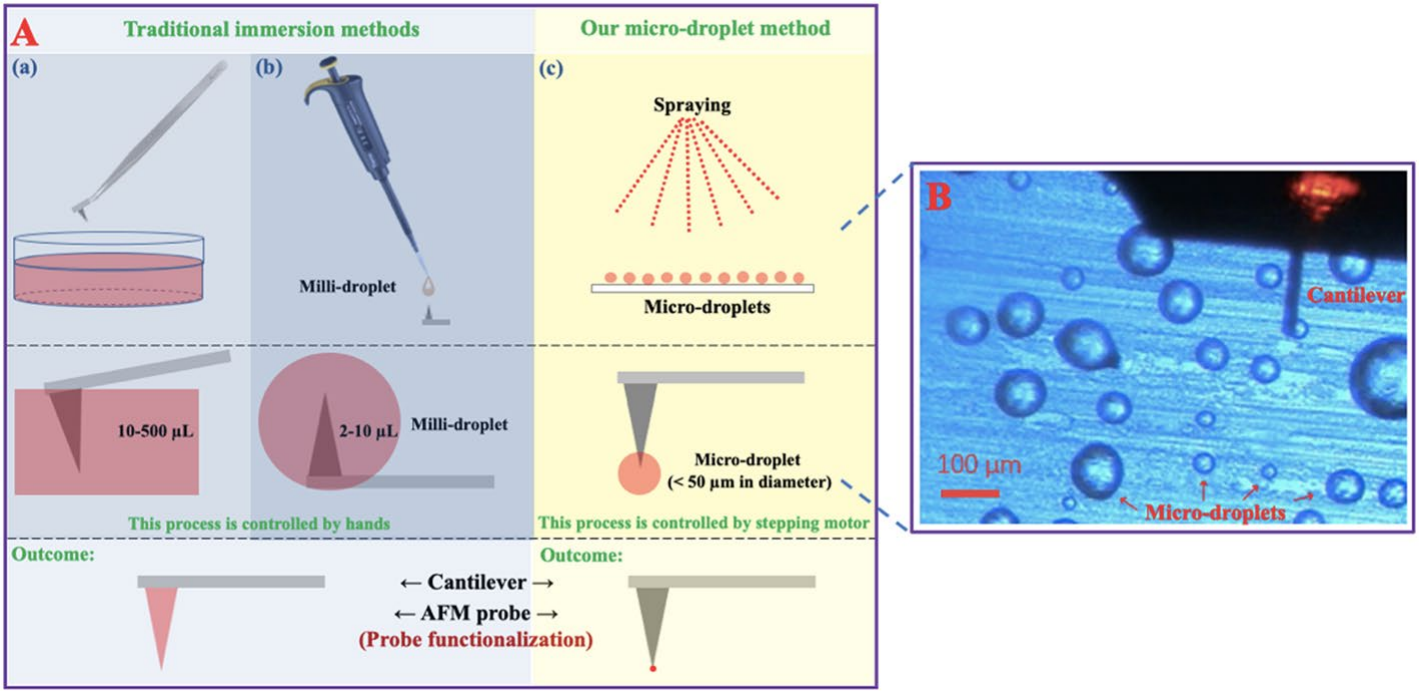
Traditional immersion methods and our novel micro-droplet method for AFM probe functionalization. **A** The schematic diagram comparing AFM probe functionalization between traditional immersion methods (a, b) and our micro-droplet method (c). From top to bottom: the main steps of each method. The process of probe functionalization is controlled by operator’s hands and a stepping motor, respectively, in traditional immersion methods and our micro-droplet method. **B** Observation of micro-droplets on a mica under an AFM cantilever/probe (even the smallest micro-droplets of up to several microns in diameter can be selected for probe functionalization)

The strength of LDL/oxLDL–LDLR/CD36 binding is another scientific question: in other words, how large are the LDL/oxLDL–LDLR/CD36 interaction forces? AFM is an ideal tool for measuring intermolecular interaction force on a piconewton scale by functionalization of the surface of an AFM probe with one of the interaction molecules. At present, two traditional methods are widely used for AFM probe functionalization, in which the entire probe is immersed in solutions (therefore, the methods are referred to as traditional immersion methods in this study; Fig. 3A-a, A-b). In the first immersion method, an AFM probe is immersed into a solution with tweezers (Fig. 3A-a), whereas in the second immersion method, a millimeter-sized drop of solution is pipetted onto an AFM probe, which is then immersed in the solution droplet (therefore, this method is referred to as a milli-droplet method here; Fig. 3A-b). These traditional immersion methods will make most of the probe surfaces functionalized with molecules, which may cause a potential force measurement of multi-molecular interactions.

To overcome this disadvantage and to improve the measurement accuracy, we develop a novel AFM probe functionalization method (Fig. 3A-c). In this method, micrometer-sized droplets (Fig. 3B) are deposited on the surface of a hydrophobic substrate (e.g., hydrophobically modified mica or glass) by spraying, and an AFM probe is controlled by a stepping motor, instead of by operator’s hands as in the immersion methods, to dip lightly into a micro-droplet (therefore, this method is referred to as a micro-droplet method). This method will make only the very tip of an AFM probe functionalized with molecules, therefore avoiding the multi-molecular interaction as much as possible. In our micro-droplet method, even the smallest micro-droplets of up to several microns in diameter can be selected for probe functionalization (Fig. 3B).

**Fig. 4:**
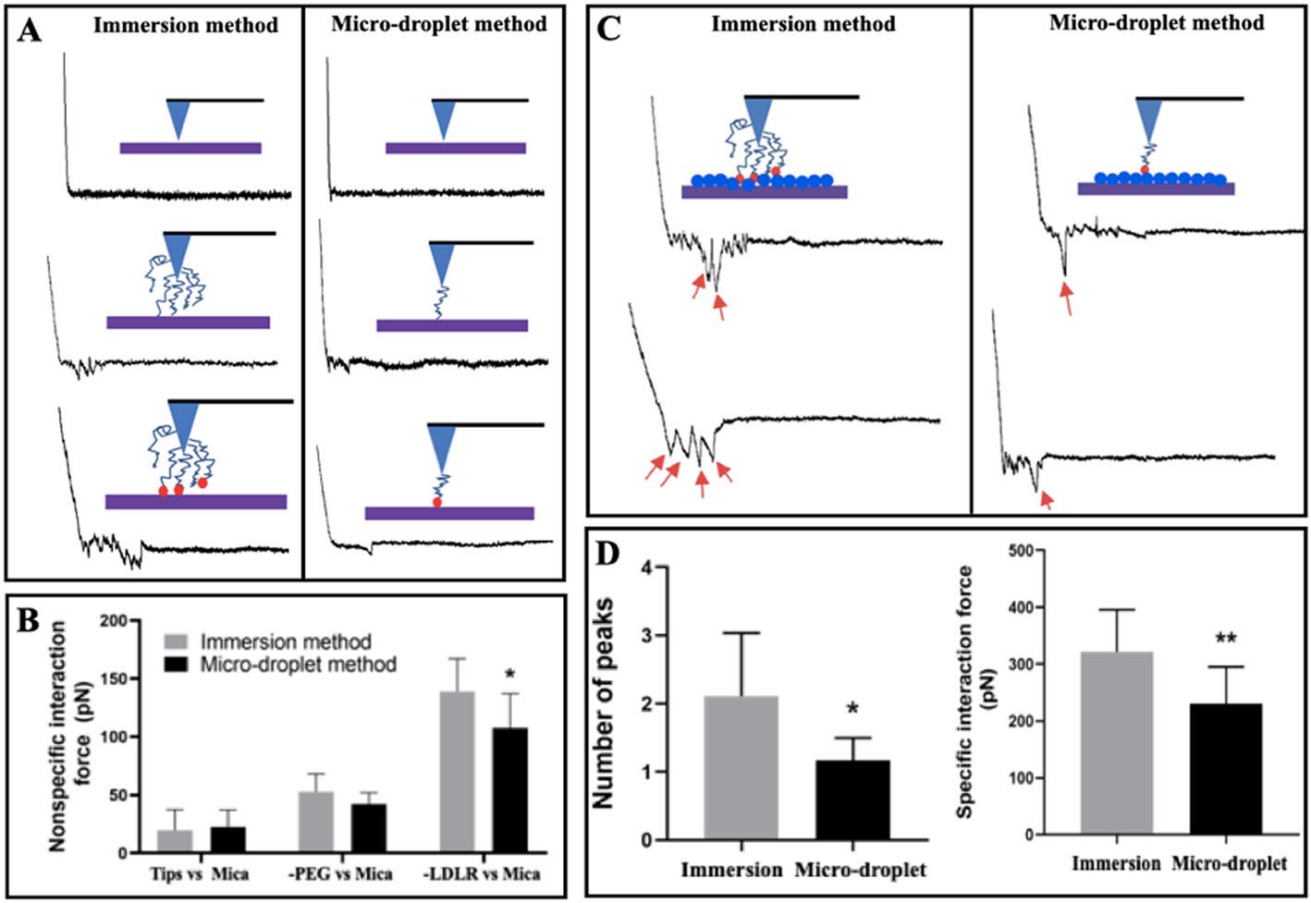
Comparison of the effects of two AFM probe functionalization methods, a traditional immersion method and our micro-droplet method, on force spectroscopic measurements. **A** Representative force spectroscopy of nonspecific interactions (force–distance curves) between bare micas and low-density lipoprotein receptors (LDLRs) functionalized on AFM probes via the immersion method (left panels) and our micro-droplet method (right panels), respectively. From top to bottom: the probes were functionalized with nothing, PEG, and PEG + LDLR, respectively. **B** Quantitative analysis of the average nonspecific interaction force. **C** Representative force spectroscopy of specific interactions between LDL particles deposited on mica and LDLRs functionalized on AFM probes via immersion method (left panels) and micro-droplet method (right panels), respectively. The red arrows on the force–distance curves show the number of interactions. **D** Quantitative analyses of the average peak number (left panel) and average force (right panel) of specific LDL–LDLR interaction. In the schematic diagrams alongside representative force–distance curves, PEG, LDLR, and LDL are displayed as a blue curly line, red dot, and blue dot, respectively (more PEG and LDLR are shown in the left panels than in the right panels). AFM force spectroscopy were performed in PBS buffer at pH 7.4. In **B** and **D**, *n* = 200 interactions in each group from three independent experiments. ^*^p < 0.05 and ^**^p < 0.01 compared our micro-droplet method with the immersion method

To test the effectiveness of our method, we utilized AFM probes functionalized with LDLR receptors via a traditional immersion method and our micro-droplet method, respectively, to measure LDLR–mica nonspecific interaction (Fig. 4A, B) and LDLR–LDL specific interaction (Fig. 4C, D). It seems that the force–distance curves obtained by AFM probes functionalized via the immersion method generally have multiple peaks (implying the multi-molecular interaction), whereas those obtained by probes functionalized via our micro-droplet method mainly have one peak (left panels of Fig. 4A, C; the red arrows indicate the peaks). Quantitative analysis also confirms the observation (average 1 and 2 peaks, respectively, for our micro-droplet method and the immersion method; left panel of Fig. 4D). Moreover, both the LDLR–mica nonspecific interaction force (Fig. 4B) and the LDLR–LDL specific interaction force (right panel of Fig. 4D) measured by AFM probe functionalized via the immersion method are significantly higher, probably due to the multi-molecular interactions, than those via our micro-droplet method. The average LDL–LDLR specific interaction force in a solution of pH 7.4 is 230.7 ± 64.5 pN for our micro-droplet method and 321.7 ± 74.0 pN for the traditional immersion method, respectively (right panel of Fig. 4D). The data imply the effectiveness and measurement accuracy of our micro-droplet method.

### The specific interaction force between native/oxidized LDL particles and corresponding receptors at various pHs detected by AFM force spectroscopy

**Fig. 5:**
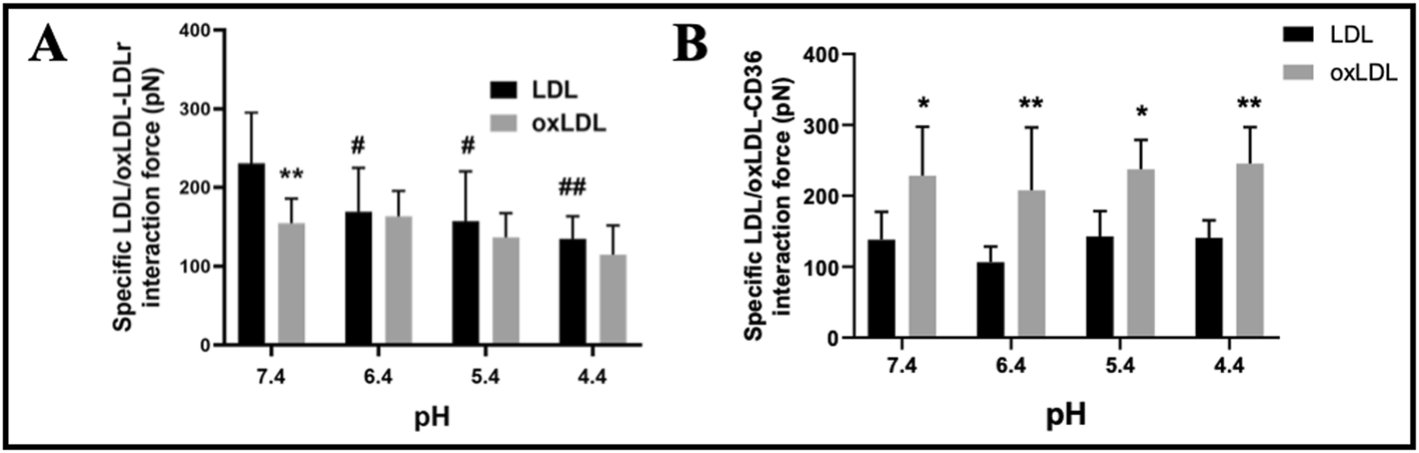
AFM force spectroscopy of specific interactions of LDL or oxLDL with LDL receptor (LDLR) or CD36 (a type of scavenger receptors) under various acidic conditions. **A** Specific interactions between LDL/oxLDL and LDLR. **B** Specific interactions between LDL/oxLDL and CD36. (*n* = 200 interactions in each group from three independent experiments; ^*^p < 0.05 and ^**^p < 0.01 compared oxLDL with LDL; ^#^*p* < 0.05 and ^##^*p* < 0.01 compared with the pH 7.4 groups). All AFM force spectroscopic experiments were performed in PBS buffer at different pH values

Next, the micro-droplet method for AFM probe functionalization was applied to answer the following scientific question: Can solution acidification influence the specific interaction force between LDL/oxLDL and their receptors? The interaction force between purified LDLR (Fig. 5A) or CD36 (Fig. 5B) functionalized on the probe tip of an AFM probe via our micro-droplet method and LDL/oxLDL particles immobilized on a mica was detected in liquid by the force spectroscopic function of AFM.

To date, there are no reports about measuring the receptor interaction force of LDL/oxLDL, although AFM force spectroscopy has been widely used to detect the interaction force between biological molecules/cells [19–21]. For the first time, the LDL/oxLDL–LDLR/CD36 interaction forces were detected by AFM force spectroscopy in this study. Under the physiological condition (i.e., at pH 7.4), the average LDL–LDLR and oxLDL–CD36 interaction forces were 230.7 ± 64.4 pN and 228.6 ± 69.1 pN, respectively (the pH 7.4 groups in Fig. 5). Since no reports support the LDL–CD36 or oxLDL–LDLR affinity, the AFM-measured average LDL–CD36 (138.0 ± 39.4 pN) and oxLDL–LDLR (154.5 ± 31.3 pN) interaction forces should be nonspecific forces. The data show that the average LDL–LDLR or oxLDL–CD36 interaction force was significantly higher than the average LDL–CD36 or oxLDL–LDLR interaction force, implying that the specific ligand–receptor interactions have a stronger force than the nonspecific LDL–CD36 or oxLDL–LDLR interactions.

Upon solution acidification, the LDL–LDLR specific interaction force significantly dropped gradually from 230.7 ± 64.4 pN (pH 7.4) down to 134.3 ± 28.9 pN (pH 4.4) with the decrease of solution pH value (the black groups in Fig. 5A), whereas the oxLDL–CD36 specific interaction force (the gray groups in Fig. 5B) and the LDL–CD36 (the black groups in Fig. 5B) or oxLDL–LDLR (the gray groups in Fig. 5A) nonspecific interaction force remained with no statistically significant changes. The data imply that solution acidification could influence the LDL–LDLR specific interaction force but not the oxLDL–CD36 specific interaction force and the nonspecific interaction forces. Of note, when pH ≤ 6.4, there was no statistically significant difference between LDL–LDLR interaction force and oxLDL–LDLR interaction force, implying that the acidification (lower than pH 6.4) could cause the loss of the LDL–LDLR specific interaction (or decrease to a level similar to nonspecific interaction).

### The interaction force between native/oxidized LDL particles and cell surfaces at various pHs measured by AFM force spectroscopy

**Fig. 6:**
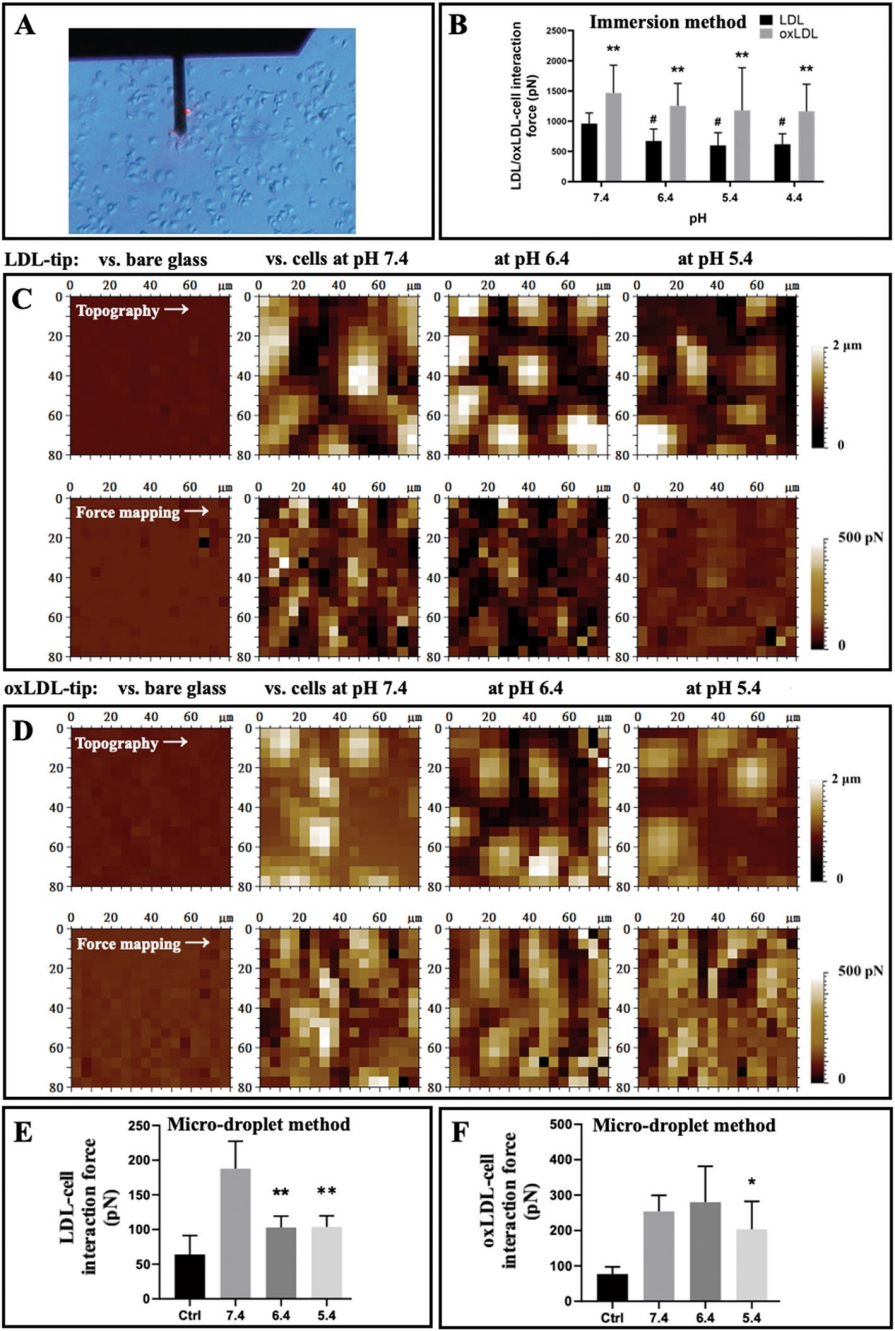
Force spectroscopy of the interactions between LDL/oxLDL particles functionalized on AFM probe tips and cell surfaces of endothelial cells under various acidic conditions. **A** Optical observation of endothelial cells under an AFM cantilever. **B** Statistical analysis of the interaction forces between LDL or oxLDL particles modified on AFM tips via the immersion method and cell surfaces under different acidic conditions. ^**^p < 0.01 compared oxLDL with LDL; ^#^*p* < 0.05 compared with pH 7.4 (*n* = 300 interactions in each group from three independent experiments). **C** The dynamic changes in topographical (top panels) and force (bottom panels) mapping of the same cells but under different acidic conditions (from left to right: pH 7.4, 6.4, and 5.4, respectively) detected by LDL-modified AFM tips via the micro-droplet method, respectively. **D** Topographical (top panels) and force (bottom panels) mapping of cells under different acidic conditions (from left to right: pH 7.4, 6.4, and 5.4, respectively) detected by oxLDL-modified AFM tips via the micro-droplet method, respectively. **E**, **F** Statistical analysis of the interaction forces between LDL (**E**) or oxLDL (**F**) particles modified on tips via the micro-droplet method and cell surfaces under different acidic conditions. The control groups represent the interactions between LDL/oxLDL-modified tips via the micro-droplet method and bare glass (i.e., the substrate of a petri dish for cell culture) at pH 7.4. ^*^p < 0.05 and ^**^p < 0.05 compared with pH 7.4 (*n* = 300 interactions in each group from three independent experiments). All AFM force spectroscopic experiments were performed in PBS buffer at different pH values

In the above experiments, the purified LDLR/CD36 receptor molecules were used for the lipoprotein–receptor interaction force measurements. Other questions may arise: What about the interaction force, and can it be influenced by solution acidification if plasma lipoproteins directly interact with receptors on cell surfaces? Therefore, AFM probes functionalized with LDL/oxLDL particles were subsequently utilized to detect the interaction forces between the lipoproteins and the surfaces of living vascular cells (Fig. 6A). The expressions of the major receptors (e.g., LDLR/CD36) for LDL/oxLDL in HUVECs were detected by Western blotting and fluorescence imaging, respectively (Fig. S4 in Supplementary Information).

Firstly, the interaction forces between LDL/oxLDL particles functionalized on AFM probes via the traditional immersion method and the surfaces of living endothelial cells were measured. We found that, by using the immersion method for AFM probe functionalization, the measured interaction forces between LDL/oxLDL and cellular surfaces are much higher (approximately 1000 pN and 1500 pN for LDL–cell and oxLDL–cell interaction forces, respectively; Fig. 6B).

Subsequently, the interaction forces between LDL/oxLDL particles functionalized on AFM probe tips via our micro-droplet method and the surfaces of living endothelial cells were mapped and measured. Figure 6C displays the topographical mapping of the endothelial cells (upper panel) and the corresponding LDL–cell force mapping (bottom panel) on the same cells after dynamically changing the solution from pH 7.4 to pH 5.4 (from left to right) at the AFM sample stage by using a simple homemade microfluidic system. Figure 6D displays the topographical mapping of the endothelial cells (upper panel) and the corresponding LDL–cell force mapping (bottom panel) on cell surfaces under various acidic conditions (from left to right: pH 7.4, pH 6.4, and pH 5.4, respectively). Just based on the force mappings, it seems that solution acidification affected the LDL–cell interaction force instead of the oxLDL–cell interaction force.

Statistical analyses show that the LDL–cell and oxLDL–cell interaction forces detected by AFM probe tips functionalized via our micro-droplet method were 187.8 ± 39.5 pN (the pH 7.4 group in Fig. 6E) and 253.8 ± 45.6 pN (the pH 7.4 group in Fig. 6F), respectively. Upon solution acidification, the LDL–cell interaction force significantly reduced to 102.9 ± 16.3 pN at pH 6.5 or 103.9 ± 15.8 pN at pH 5.4 (Fig. 6E), whereas the oxLDL–cell interaction force had no statistically significant change at pH 6.4 (280.1 ± 101.5 pN) but also significantly decreased to 203.5 ± 78.7 pN at pH 5.4 (Fig. 6F). The data imply that the oxLDL–cell interaction was more tolerant of solution acidification than the LDL–cell interaction and that the oxLDL–cell interaction force was much higher than the LDL–cell interaction force under acidic conditions.

### Acidification affects the LDL–LDLR binding/recognition by influencing both LDL and LDLR

To determine which side of the LDL–LDLR interaction pair was influenced by acidification, two additional experiments were designed: (1) Native LDL particles were incubated in solution at different pHs (pH 7.4, pH 6.4, and pH 4.4, respectively) at 37 °C for ~1 h, and then interacted with LDLR molecules pre-immobilized on mica at pH 7.4 at 37 °C for ~0.5 h, gently washed with PBS, and imaged by AFM in PBS at pH 7.4 at 37 °C; (2) LDLR molecules pre-immobilized on mica were incubated in solution at different pHs (pH 7.4, pH 6.4, and pH 4.4, respectively) at 37 °C for ~1 h, washed with PBS at pH 7.4, interacted with added LDL particles at pH 7.4 at 37 °C for ~0.5 h, gently washed with PBS, and then imaged by AFM in PBS at pH 7.4 at 37 °C.

**Fig. 7:**
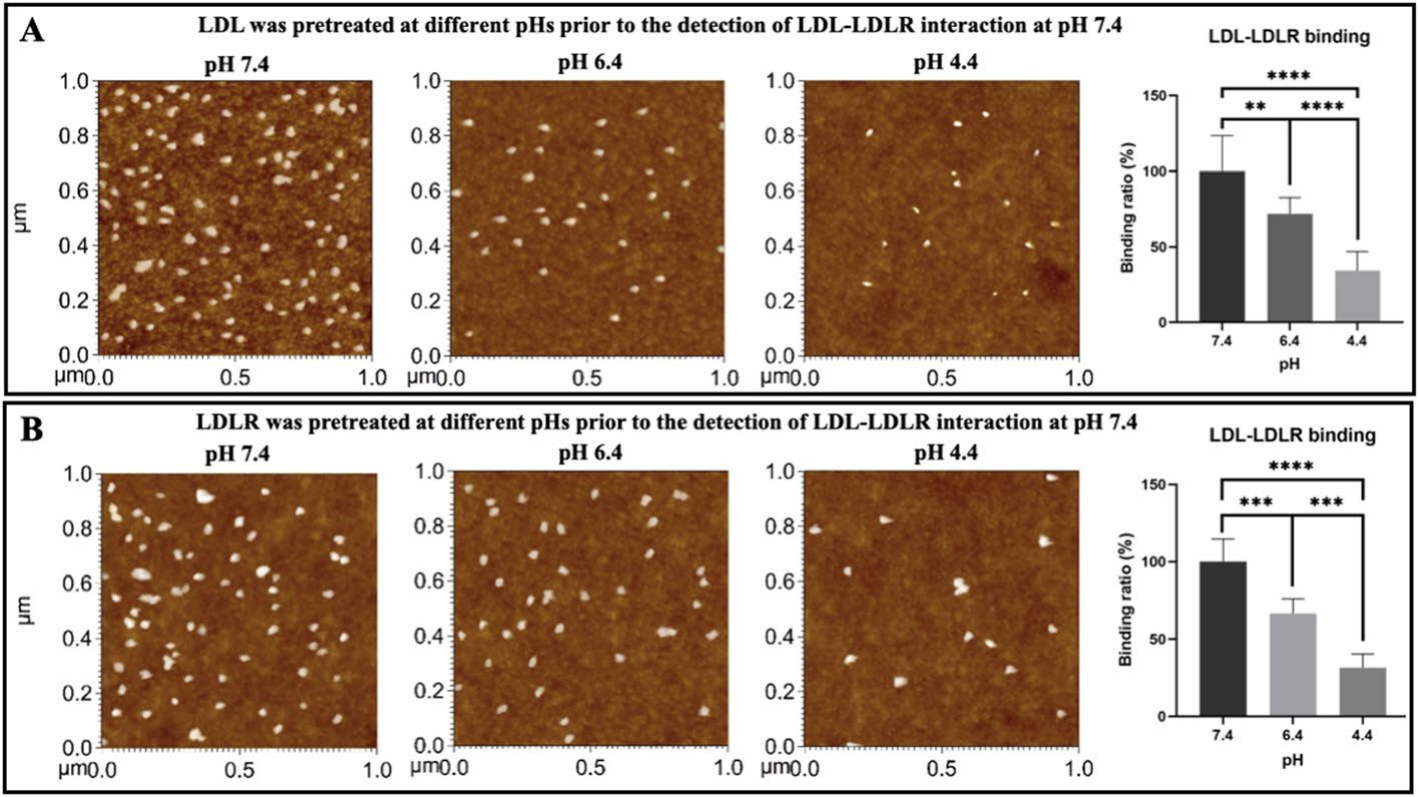
AFM topographical observation of the recognition of native LDL by the LDL receptor (LDLR) at pH 7.4, one of which was pretreated at different pHs. **A** LDL particles were treated at different pHs (from left to right: pH 7.4, pH 6.4, and pH 4.4, respectively; the rightmost panel: the statistical quantification of LDL–LDLR binding ratio) prior to AFM detection of LDL–LDLR interaction at pH 7.4. **B** LDL receptors (LDLRs) were treated at different pHs (from left to right: pH 7.4, pH 6.4, and pH 4.4, respectively; the rightmost panel: the quantification of LDL–LDLR binding ratio) prior to AFM detection of LDL–LDLR interaction at pH 7.4. ^**^p < 0.01, ^***^p < 0.001, and ^****^p < 0.0001 (*n* ≥ 5 images in each group)

Figure 7 presents the experimental results. In both experiments, LDL particles with a relatively larger size on a layer of LDLR with a small size were observed by AFM. In the first experiment in which LDL particles were incubated at different pHs prior to LDL–LDLR interaction and AFM imaging at pH 7.4, solution acidification caused an obvious decrease in particle size of LDLs, particularly at pH 4.4 (AFM topographical images in Fig. 7A), whereas no changes in LDL particle size occurred in the second experiment in which LDL receptors (LDLR) rather than LDL particles were treated by acidification (AFM topographical images in Fig. 7B). The acidification-induced size change of LDL particles coincides with our AFM and TEM observations in Figs. 1 and 2. AFM topographical images and statistical quantification show that the number of bound LDL particles or the LDL–LDLR binding ratio significantly decreased with the drop of solution pH from 7.4 to 4.4 in both experiments (Fig. 7), suggesting that both sides of the LDL–LDLR interaction pair could be influenced by acidification, which finally influenced LDL–LDLR interaction.

## Discussion

### Novel findings

In this study, we obtained some interesting and important findings. Firstly, by using the nanoscale imaging function of AFM (Fig. 1) and immuno-TEM (Fig. 2), the LDL–LDLR binding ratio was detected to significantly decrease upon solution acidification, even at pH 6.4, whereas the oxLDL–CD36 binding ratio had a statistically significant changes until the solution reached a relatively high acidification (at pH 4.4), implying that the LDL–LDLR recognition is relatively susceptible to solution acidification, whereas the oxLDL–CD36 recognition is relatively tolerant of solution acidification. The acidification-induced decrease in the LDL–LDLR binding/recognition has been discovered previously via other methods [22–24]. In our present study, however, atomic force microscopy was for the first time utilized to verify the influence of acidification on the LDL–LDLR binding/recognition. Multiple factors, including lipid composition, particle size, and the content of specific apolipoproteins (e.g., apoE) of LDL and others (e.g., electrostatic potential/interaction), have been reported to affect the receptor binding or recognition of LDL [23–26]. It is possible that solution acidification might indirectly influence the LDL–LDLR binding/recognition by regulating other direct factors e.g., the composition, particle size, and the content of specific apolipoproteins. Actually, the acidification-induced size decrease of LDL particles was observed in the present study and our previous study [11].

Secondly, we developed a novel micro-droplet method (Fig. 3) for AFM probe functionalization of receptor proteins or lipoproteins, in which the AFM probe tip can be controlled by the piezoelectric ceramic stepping motor equipped with an AFM instrument to dip lightly into the functionalizing solutions, making only the very tip of an AFM probe (instead of the entire probe/tip) functionalized with functionalizing molecules. By using this AFM probe-functionalizing method, the multi-molecular interactions can be avoided as much as possible, and more accurate measurements (significantly lower average LDLR–mica nonspecific interaction forces and a significantly lower average LDLR–LDL specific interaction force) of specific lipoprotein–receptor interaction can be achieved in comparison with a traditional immersion method (Fig. 4). This novel micro-droplet method can be a versatile method for the AFM probe tip functionalization of all samples including biological, chemical, and even polymeric molecules. The disadvantages of the novel micro-droplet method for AFM probe functionalization are the need for an experienced AFM operator and the relatively high cost due to AFM usage. However, it is not a problem for the research groups who possess AFM equipment.

**Fig. 8:**
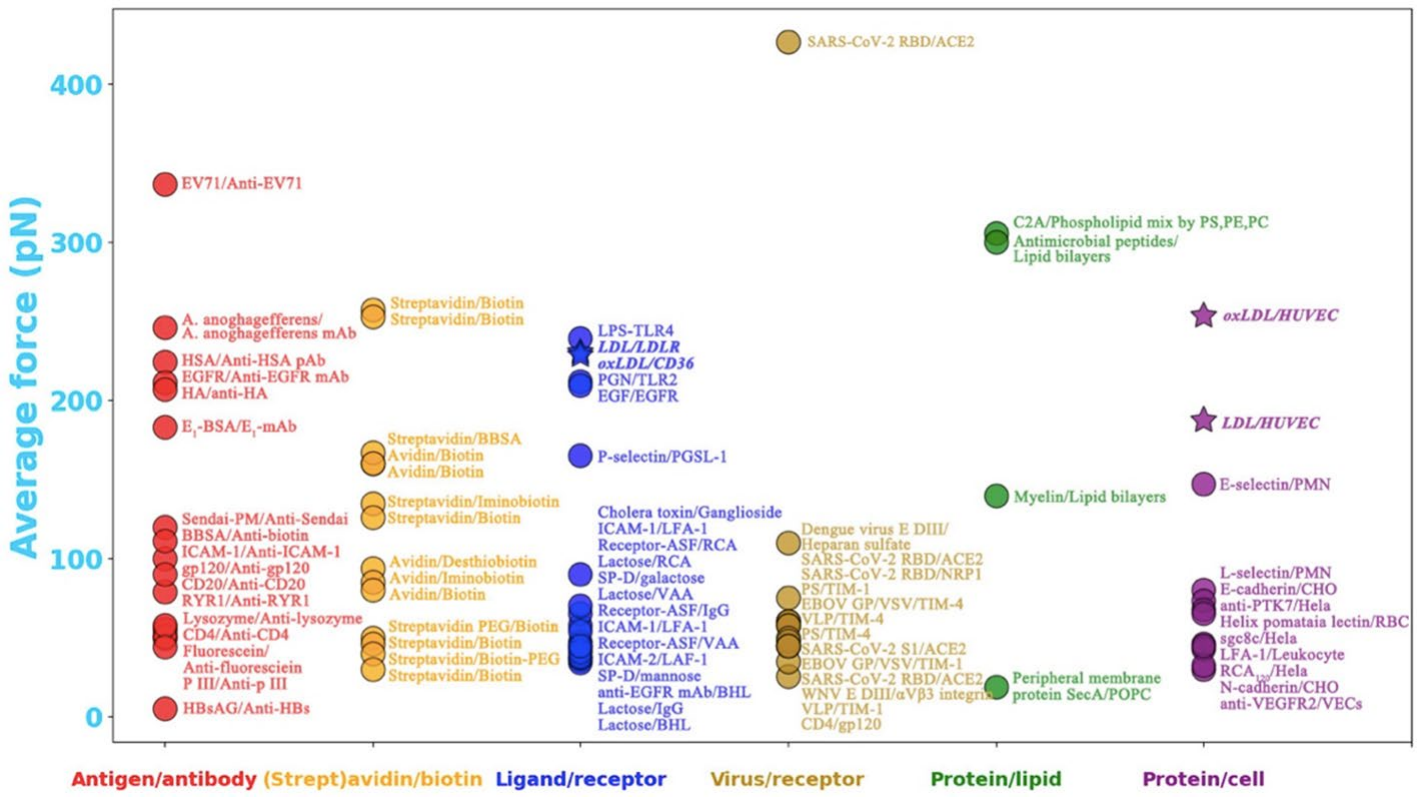
Average interaction forces (or unbinding forces) between various intermolecular interactions in literature and in the present study. The intermolecular interactions were artificially divided into six subclasses. The LDL–LDLR, oxLDL–CD36, LDL-HUVEC, and oxLDL-HUVEC interactions detected in the present study are also added into the ligand–receptor and protein–cell interaction subclasses, respectively, and were highlighted with the asterisks. EV71, enterovirus 71; *A. anophagefferens*, *Aureococcus anophagefferens*; HSA, human serum albumin; mAb, monoclonal antibody; pAb, polyclonal antibody; EGFR, epidermal growth factor receptor; EGF, epidermal growth factor; HA, hemagglutinin; E_1_-BSA, estrone–bovine serum albumin; Sendai-PM, Sendai–purple membrane; pIII, gene III protein; HBsAg, hepatitis B surface antigen; PEG, polyethylene glycol; LPS, lipopolysaccharides; PGN, peptidoglycan; ICAM-1, intercellular adhesion molecules-1; ICAM-2, intercellular adhesion molecules-2; LFA-1, leukocyte function-associated antigen-1; RCA, ricinus communis; VAA, viscum album; IgG, immunoglobulin G; BHL, bovine heart; SP-D, surfactant protein D; RBD, receptor-binding domain; ACE2, angiotensin-converting enzyme 2; NRP1, neuropilin-1; WNV, West Nile virus; PS, phosphatidylserine; PE, phosphatidylethanolamine; PC, phosphatidylcholine; EBOV, Ebola virus; VLP, virus-like particle; VSV, vesicular stomatitis virus; POPC, 1-palmitoylv-2-oleoyl-sn-glycero-3-phosphocholine; HUVEC, human umbilical vein endothelial cell; PMN, polymorphonuclear leukocyte; CHO, Chinese hamster ovary cell; RCA_120_, *Ricinus communis* agglutinin-120; VECs, vascular endothelial cells

Thirdly, for the first time, we determined the average LDL–LDLR and oxLDL–CD36 specific interaction forces, which are 230.7 ± 64.4 pN and 228.6 ± 69.1 pN, respectively, under the physiological condition (i.e., at pH 7.4; the pH 7.4 groups in Fig. 5). The AFM measured stronger forces of the LDL–LDLR and oxLDL–CD36 specific interactions (230.7 ± 64.4 pN and 228.6 ± 69.1 pN, respectively) than the LDL–CD36 and oxLDL–LDLR nonspecific interactions (138.0 ± 39.4 pN and 154.5 ± 31.3 pN, respectively) can support the common knowledge of LDLR and CD36 as the major receptors for native LDL and oxLDL, respectively. Surprisingly but interestingly, however, there is no significant difference between the average LDL–LDLR interaction force (230.7 ± 64.4 pN) and the average oxLDL–CD36 interaction force (228.6 ± 69.1 pN) under the physiological condition. By comparing some previously reported interaction forces (or unbinding forces) of other biological interaction pairs including antigen–antibody [27–42], (strept)avidin–biotin [43–51], ligand–receptor [36, 52–58], virus–receptor [29, 59–63], protein–lipid [64–67], and protein–cell [58, 68–73] interaction pairs (see references for detailed information), the LDL–LDLR and oxLDL–CD36 interaction forces are in the group with a relatively high force value, and the interaction forces of most interaction pairs are below 200 pN (Fig. 8). LDL and oxLDL particles are the complexes of lipids (e.g., phospholipids and cholesterol) and apolipoproteins (e.g., apoB-100, apoE, and other exchangeable apolipoproteins). Multiple components of LDL/oxLDL particles including lipids [74, 75] and apolipoproteins (e.g., apoB and apoE) [76, 77] have been reported to be able to interact with LDL/oxLDL receptors [78], which may partially contribute to their relatively high LDL/oxLDL–LDLR/CD36 interaction forces.

We also measured the average LDL–cell and oxLDL–cell interaction forces, which are 187.8 ± 39.5 pN (the pH 7.4 group in Fig. 6E) and 253.8 ± 45.6 pN (the pH 7.4 group in Fig. 6F), respectively, under the physiological condition (pH 7.4; Fig. 8). Surprisingly, the LDL–cell interaction force is significantly weaker than the LDL–LDLR interaction force, whereas the oxLDL–cell interaction force is higher than the oxLDL–CD36 interaction force. The most likely reason is the complicated components of a cell surface, which is composed of specific receptors for LDL/oxLDL (e.g., LDLR and CD36), nonspecific receptors (e.g., other scavenger receptor types and other non-scavenger receptors, which probably can interact weakly with LDL/oxLDL), and nonreceptor molecules (e.g., lipids, proteins, and glycolipids or glycoproteins). Naturally, free lipoprotein molecules will be automatically recognized in liquid by their corresponding receptors. In the AFM force spectroscopic experiments, lipoprotein molecules were bound on a AFM probe tip and artificially controlled to interact with molecules on a cell surface. There are probably several types of interactions, including the LDL–LDLR or oxLDL–CD36 specific interaction, the specific interactions of LDL/oxLDL with other scavenger receptors (e.g., both CD36 and LOX-1 can specifically recognize oxLDL), the nonspecific interactions of LDL/oxLDL with other receptors (e.g., LDL versus CD36 and oxLDL versus LDLR), and the nonspecific interactions (e.g., electrostatic interaction) of LDL/oxLDL with nonreceptor molecules. It has been previously reported that oxLDL has a higher nonspecific adhesive force (or stickiness) than native LDL [18]. In this study we found that the oxLDL–LDLR nonspecific interaction (154.5 ± 31.3 pN) has a higher average force than the LDL–CD36 nonspecific interaction (138.0 ± 39.4 pN) under the physiological condition (the pH 7.4 groups in Fig. 5). Moreover, apart from CD36, oxLDL can be specifically recognized by other scavenger receptors, including SR-AI/II, SR-BI, and LOX-1, which also are highly expressed on vascular cells (e.g., endothelial cells, macrophages, and smooth muscle cells) [79–82]. These data may partially support the higher oxLDL–cell interaction force than the LDL–cell interaction force.

Fourthly, also for the first time, we determined the influence of solution acidification on the LDL/oxLDL–receptor interaction force. The lipoprotein–receptor AFM force spectroscopy revealed that only the LDL–LDLR specific interaction force at an acidic pH (e.g., pH 6.4–pH 4.4) had a significant decrease compared with the pH 7.4 group, whereas the oxLDL–CD36 specific interaction force and the LDL–CD36 or oxLDL–LDLR nonspecific interaction forces had no changes upon solution acidification (Fig. 5). Similarly, the lipoprotein–cell AFM force spectroscopy revealed that the LDL–cell interaction force could be significantly reduced even at pH 6.4, whereas the oxLDL–cell interaction force significantly decreased under a more acidic condition, i.e., at pH 5.4 (Fig. 6). These data imply that the LDL–LDLR and LDL–cell interactions are relatively susceptible to solution acidification, whereas other interactions including oxLDL–CD36 and oxLDL–cell interactions are relatively tolerant of solution acidification. It is unclear why solution acidification could cause the changes in LDL–LDLR interaction force and the differential effects on LDL and oxLDL. For LDL–LDLR interaction, we found that acidification could influence the interaction by changing both sides of the LDL–LDLR interaction pair (Fig. 7). However, the underlying molecular mechanisms might be complicated and cannot be uncovered presently. Actually, several different molecular mechanisms have been proposed for LDL binding at neutral pH to LDLR and LDL release at acidic pH from LDLR, among which the structural changes of LDLR and apolipoproteins (e.g., apoB and apoE) at acidic pH are potentially major reasons [76, 77, 83, 84]. Therefore, the changes in both the LDLR structure and the apolipoprotein structure/content in LDL particles may be responsible for the acidification-induced changes in the LDL–LDLR and/or oxLDL–CD36 interaction forces. Moreover, it is unclear whether the effect of acidification on the LDL–LDLR interaction was also attributed to the changes in the lipid composition of LDL particles. To further address this possibility, more in-depth studies will be needed in the future.

### Biomechanical/physiological/pathological implications

**Fig. 9:**
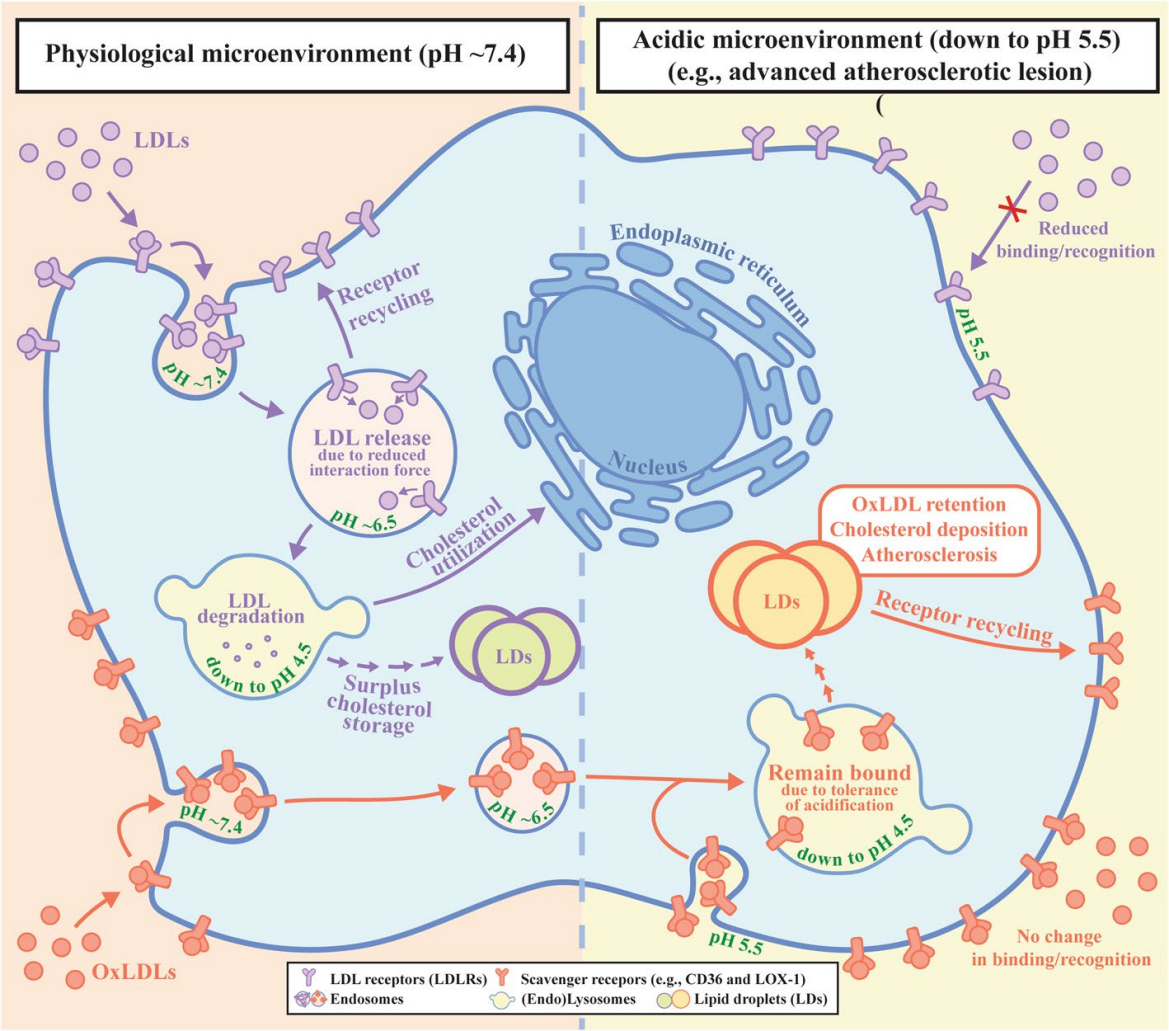
Schematic diagram presenting the biomechanical/physiological/pathological implications of our findings. Differential influences of microenvironmental/lysosomal solution acidification on receptor binding/recognition and interaction force of different LDL forms (e.g., native and oxidized LDLs) induce their different fates and atherosclerosis

Our findings have two major aspects of biomechanical/physiological/pathological implications (Fig. 9). Firstly, at the cell surface, the extracellular microenvironment acidification down to pH ~5.5 in specific tissues (e.g., advanced atherosclerotic plaques) will lower the binding/recognition between LDL particles and LDLR, whereas the binding/recognition between modified LDL particles (e.g., oxLDL) and scavenger receptors (e.g., CD36) has no significant change. The differential influences of acidification on the receptor recognitions of LDL and oxLDL particles at the cell surface will let cells take up more oxLDL particles but fewer native LDL particles. Moreover, the significantly smaller effect of acidification may make the CD36-mediated oxLDL uptake unsusceptible to cholesterol levels. Secondly, during cellular endocytosis, the endosomal acidification from pH 7.4 down to pH 4.4 will significantly lower the interaction force between LDL particles and LDLR at pH ≤ 6.5, whereas the interaction force between oxLDL particles and scavenger receptors (e.g., CD36) has no significant change until reaching a relatively strong acidic condition (e.g., at pH ≤ 4.4). The influence of acidification on the LDL–LDLR interaction force may cause a relatively easy release of LDL particles from LDLRs even in early endosomes (an intracellular microenvironment of pH ~6.5) for further degradation of individual LDL particles in endolysosomes or lysosomes (pH ~4.5). On the contrary, no significant effect of acidification on the oxLDL–receptor interaction force will make oxLDL particles remain bound to the receptors at the inner side of the (endo)lysosomal membrane and subsequently the oxLDL–receptor complexes go into lipid droplets (LDs) [85]. Therefore, oxLDL particles cannot be further degraded easily, but may induce oxLDL retention, lipid deposition in cells, foam cell transformation, and finally atherosclerosis.

It is worth mentioning that the influence of acidification on LDL–LDLR interaction mainly limits at the relatively early stages of atherosclerotic development because in advanced atherosclerotic lesions the expression of LDLR becomes relatively low. On the contrary, the expression of scavenger receptors (e.g., CD36 for oxLDL) significantly increases in advanced atherosclerotic lesions. Therefore, the resistance of oxLDL–receptor interaction to acidification will become more relevant and more important for atherogenesis.

## Conclusions

In this study, by using the nanoscale imaging function of atomic force microscopy (AFM) and immuno-transmission electron microscopy (immuno-TEM), we verified the acidification-induced decrease in the LDL–LDLR binding/recognition, which was previously reported, and also found the tolerance of the oxLDL–CD36 binding/recognition of solution acidification. By developing a novel, versatile, micro-droplet-based method for AFM probe functionalization of receptors or lipoprotein particles, the force spectroscopic function of AFM was applied to detect the LDL/oxLDL–LDLR/CD36 interaction forces on mica and on cell surfaces, respectively, in liquid at different pHs. For the first time, we determined the LDL–LDLR, oxLDL–CD36, LDL–cell, and oxLDL–cell interaction forces, and found that the LDL–LDLR and LDL–cell interaction forces are relatively susceptible to solution acidification, whereas the oxLDL–CD36 and oxLDL–cell interaction forces are relatively tolerant of solution acidification.

The susceptibility of the LDL–LDLR/cell interaction force to solution acidification implies that the intracellular lysosomal acidification may cause the relatively easy release of LDL particles from the membrane-bound receptors (i.e., LDLR) into the lysosomal solution for LDL degradation. The susceptibility of the LDL–LDLR binding/recognition to acidification and the acidification tolerance of the oxLDL–CD36 binding/recognition imply the superiority of oxLDL uptake in an acidic microenvironment in advanced atherosclerotic plaques. Moreover, the acidification tolerance of the oxLDL–CD36/cell interaction force implies that it is hard for the intracellular lysosomal acidification to cause the release of oxLDL particles for degradation, which may trigger and/or enhance lysosomal oxLDL retention, intracellular lipid deposition, foam cell transformation, and finally atherogenesis. Therefore, our data provide important information and biomechanical/pathological implications for understanding lysosomal LDL/oxLDL degradation and particularly atherogenesis induced by modified LDL (e.g., oxLDL).

## Supplementary Information

Additional file 1.

## Data Availability

All data generated or analyzed during this study are included in this study, and the original data are available upon request.

## Abbreviations

AcLDL: Acetylated low-density lipoprotein
AFM: Atomic force microscopy
APTES: Aminopropyltrethoxysilane
ASCVD: Atherosclerotic cardiovascular diseases
CM: Chylomicron
CVD: Cardiovascular diseases
DIPEA: *N*,*N*-Diisopropylethylamine
EDC: 1-Ethyl-3-(3-dimethylaminopropyl) carbodiimide
FBS: Fetal bovine serum
HDL: High-density lipoprotein
HDL-C: High-density-lipoprotein cholesterol
HEPES: Hydroxyethyl piperazine ethanesulfonic acid
HUVECs: Human umbilical vein endothelial cells
IDL: Intermediate-density lipoprotein
LD: Lipid droplet
LDL: Low-density lipoprotein
LDL-C: Low-density-lipoprotein cholesterol
LDLR: Low-density lipoprotein receptor
MDA: Malondialdehyde
MES-PEG-NHS: *N*-Hydroxysuccinimide-polyethylene glycol-mesylate
MPTMS: Mercaptopropyltrimethoxysilane
oxLDL: Oxidized low-density lipoprotein
PBS: Phosphate-buffered saline
sdLDL: Small dense low-density lipoprotein
SR-B1: Class B scavenger receptor type 1
SRs: Scavenger receptors
TBARS: Thiobarbituric acid reactive substances
TCEP: Trichloroethyl phosphate
TEM: Transmission electronic microscopy
VLDL: Very-low-density lipoprotein
