# Supplementary material for: AFM reveals differential effects of acidification on LDL– and oxidized LDL–receptor interactions: biomechanical implications in atherogenesis
Source: Cell Mol Biol Lett. 2025 Mar 18;30:32. doi: 10.1186/s11658-025-00715-9 (PMC11921551; doi:10.1186/s11658-025-00715-9)
Supplement: Supplementary file 1 — Additional file 1. [file 11658_2025_715_MOESM1_ESM.docx]

**Supplementary Information**

**AFM reveals differential effects of acidification on LDL- and oxidized LDL-receptor interactions: Biomechanical implications in atherogenesis**

Kun Wang^1,2^, Chenhan Sun^1^, Hongda Zhuang^1^, Xian-Cheng Jiang^3^, Yong Chen^1,2,4,*^

^1^ Institute for Advanced Study, Nanchang University, Nanchang, Jiangxi330031, China

^2^ School of Life Sciences, Nanchang University, Nanchang, Jiangxi330031, China

^3^ Department of Cell Biology, SUNY Health Science University, State University of New York, Brooklyn, NY 11203, USA

^4^ School of Pharmacy, Nanchang University, Nanchang, Jiangxi330031, China

*** Corresponding author:**

Mailing address: 999 Xuefu Ave., Honggutan District, Nanchang, Jiangxi 330031, P. R. China

Tel/fax: 86-791-83969963

Email: tychen@ncu.edu.cn or dr_yongchen@hotmail.com

**Running title:** LDL/oxLDL-receptor interactions at various pHs

**Table of Contents**

**Fig. S1.** Cell viability of HUVECs under the indicated acidic conditions for 6 h.

**Fig. S2.** Representative AFM topographical images of oxLDL particles binding on a CD36 receptor layer under different acidic conditions.

**Fig. S3.** Representative immune-TEM images of gold nanoparticle (5 nm)-conjugated CD36 receptors specifically binding on oxLDL particles under different acidic conditions.

**Fig. S4.** Expressions of LDLR and CD36 in HUVEC cells in media supplemented with 10%, 1%, and 0% FBS, respectively detected by western blotting and fluorescence imaging.


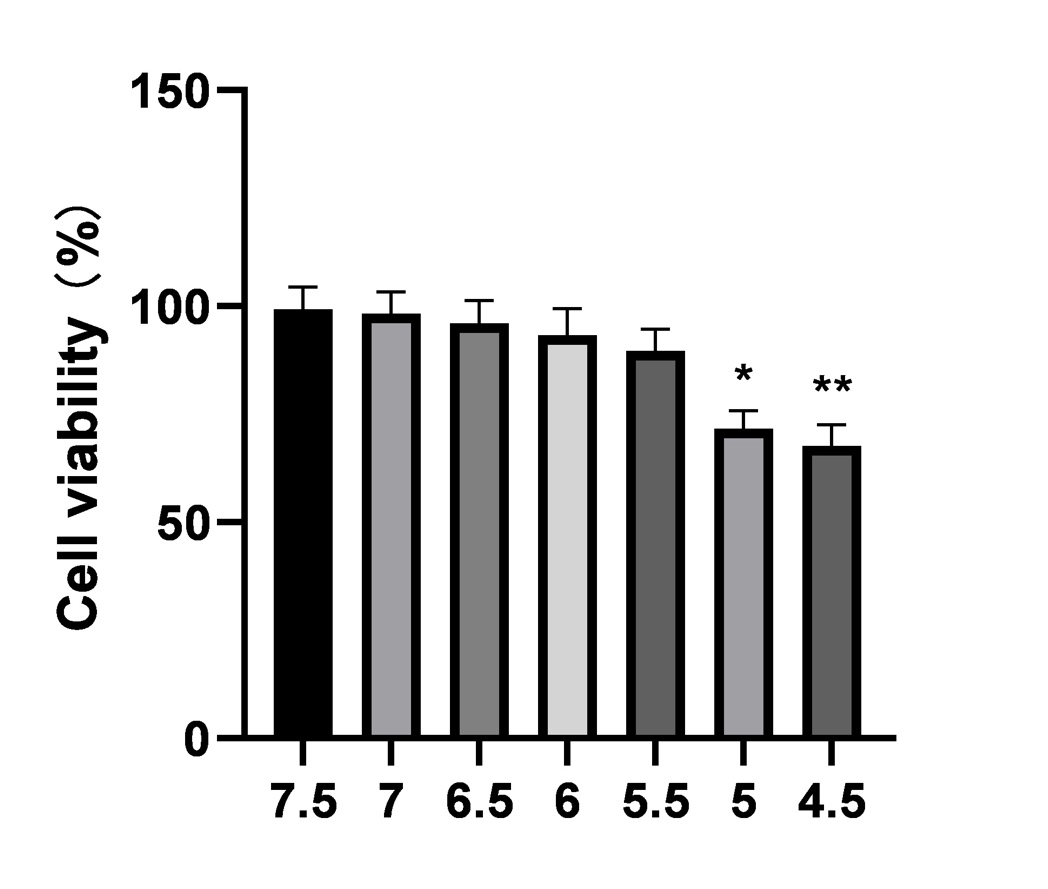


**Fig. S1. Cell viability of HUVECs under the indicated acidic conditions for 6 h detected by MTT assay.** * p < 0.05 and ** p < 0.01 compared with pH 7.5.


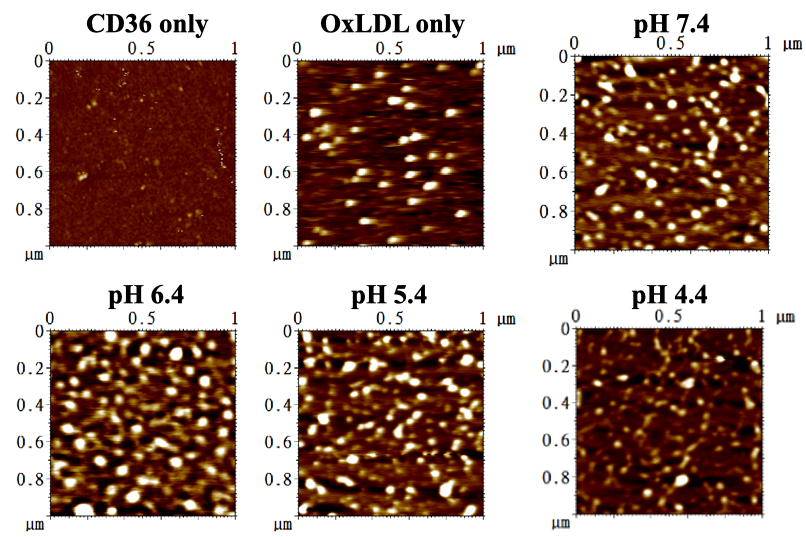


**Fig. S2. Representative AFM topographical images of oxLDL particles binding on a CD36 receptor layer under different acidic conditions.**


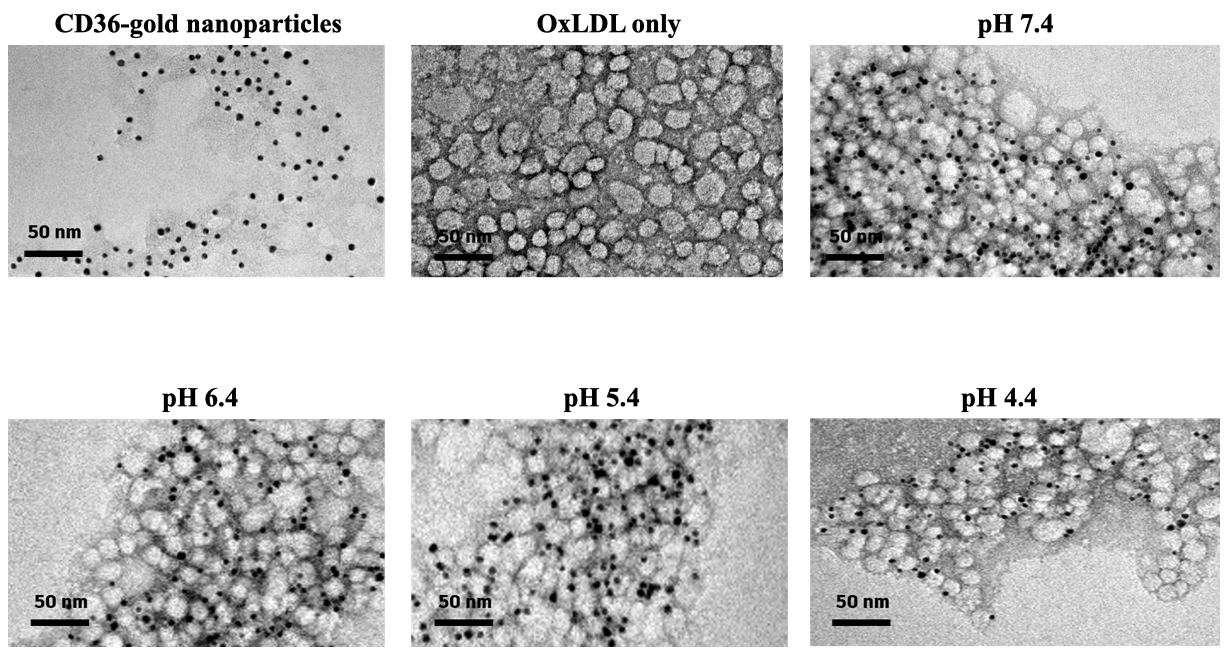


**Fig. S3. Representative immune-TEM images of gold nanoparticle (5 nm)-conjugated CD36 receptors specifically binding on oxLDL particles under different acidic conditions.**

**B**

**A**


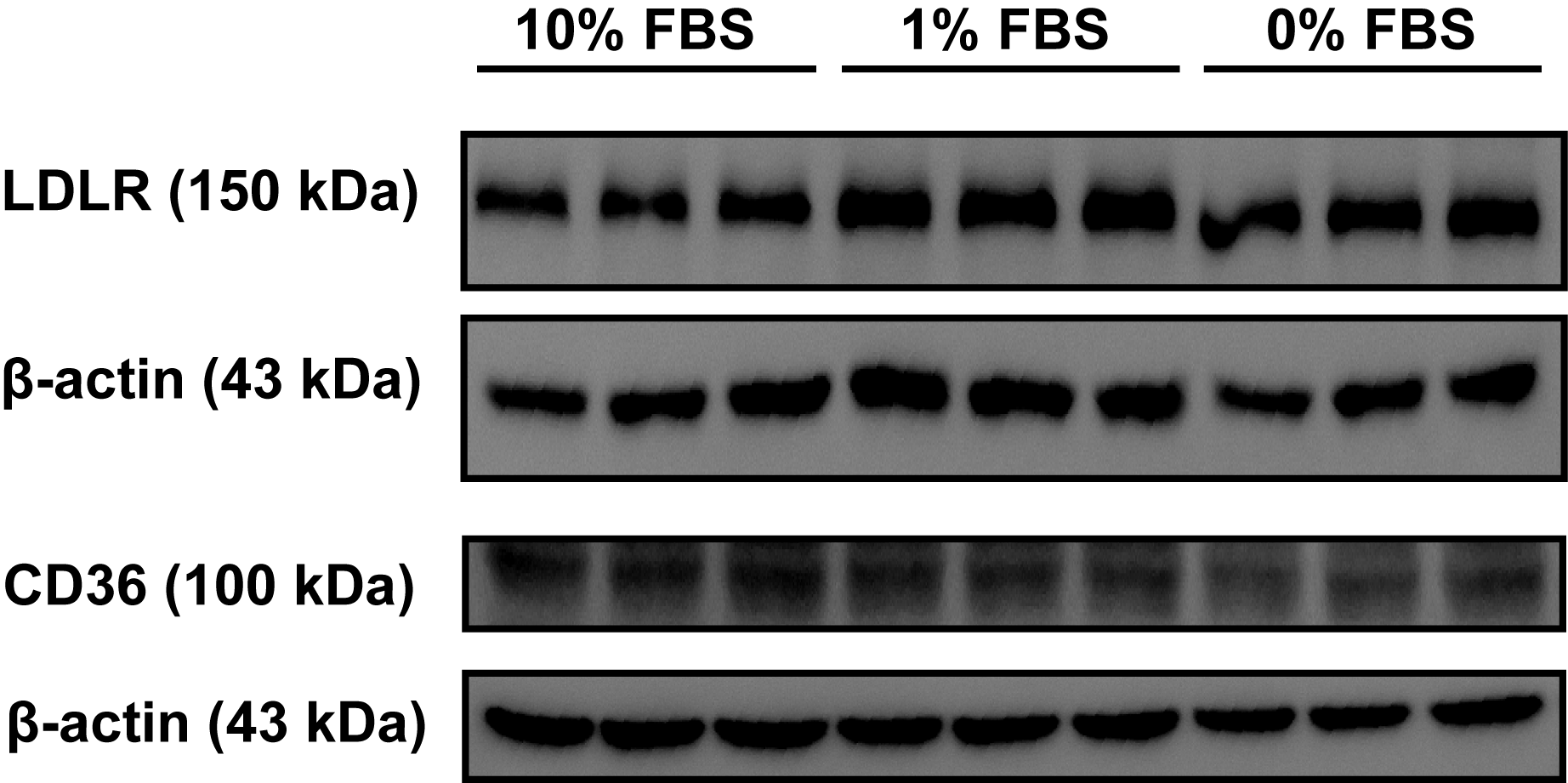


**D**

**C**


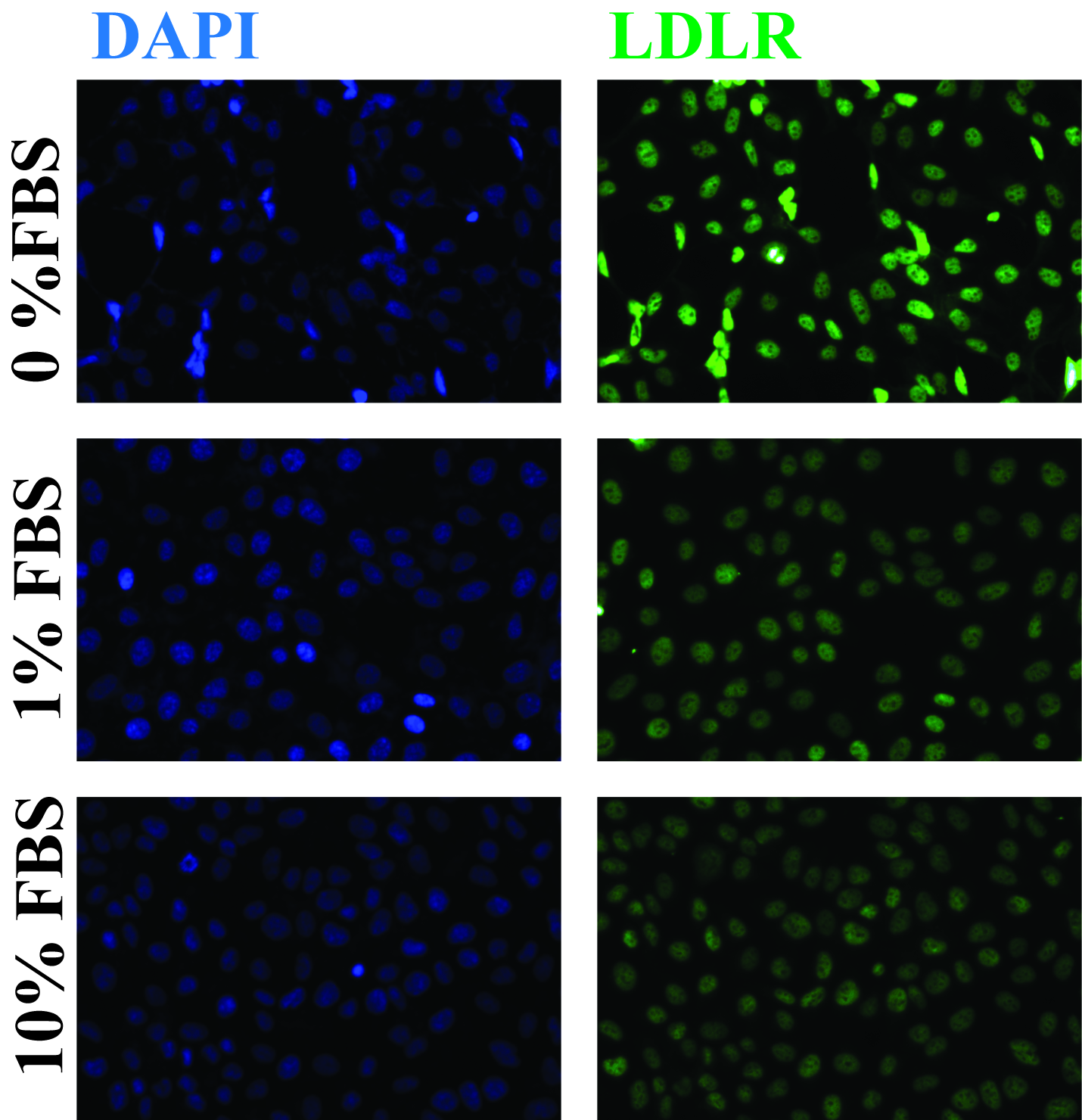

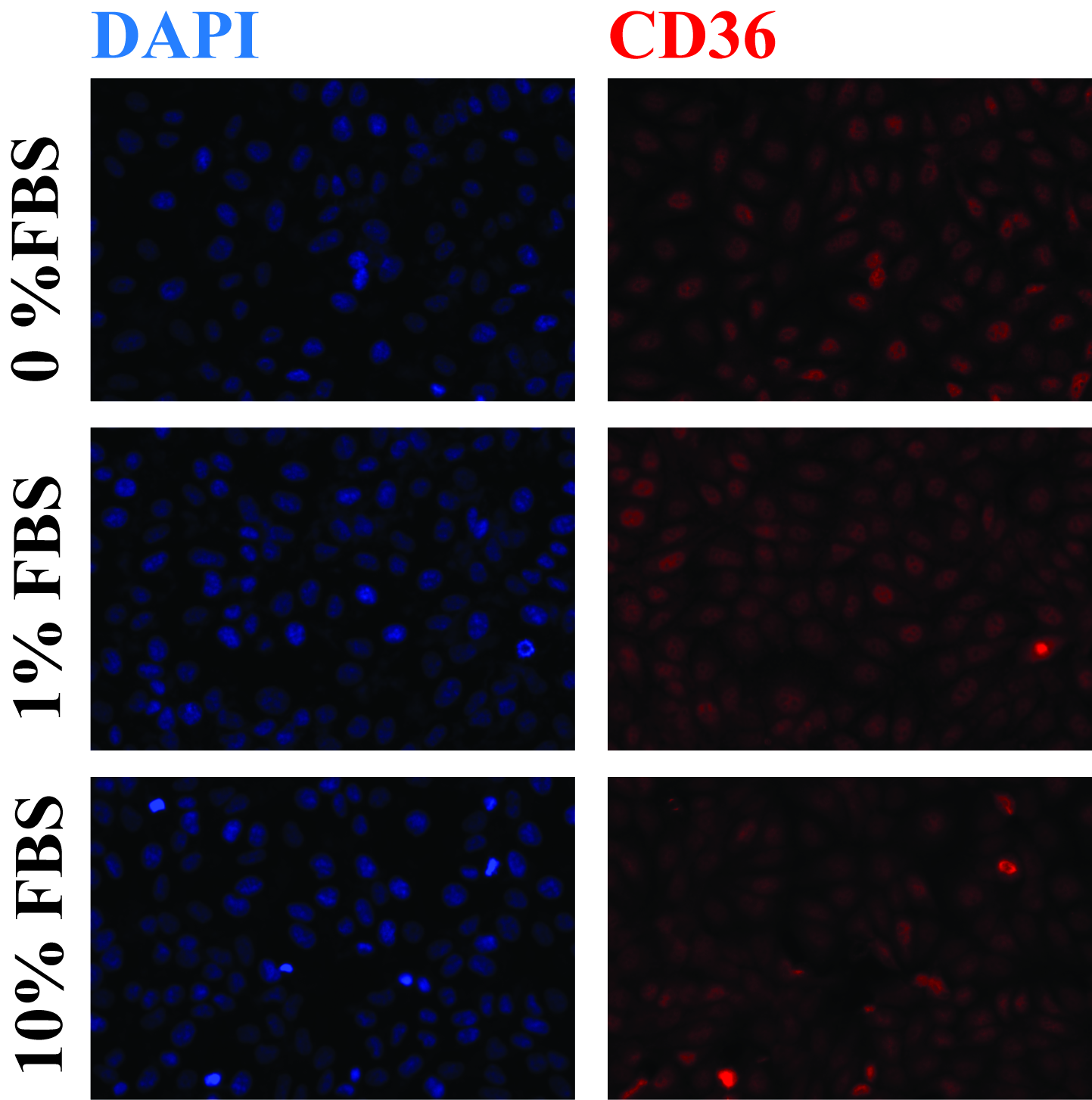


**Fig. S4. Expressions of LDLR and CD36 in HUVEC cells in media supplemented with 10%, 1%, and 0% FBS, respectively detected by western blotting (A, B) and fluorescence imaging (C, D).**
